# Ecophysiological Study of *Paraburkholderia* sp. Strain 1N under Soil Solution Conditions: Dynamic Substrate Preferences and Characterization of Carbon Use Efficiency

**DOI:** 10.1128/AEM.01851-20

**Published:** 2020-11-24

**Authors:** K. Taylor Cyle, Annaleise R. Klein, Ludmilla Aristilde, Carmen Enid Martínez

**Affiliations:** a Soil and Crop Sciences, School of Integrative Plant Science, College of Agriculture and Life Sciences, Cornell University, Ithaca, New York, USA; b Department of Biological and Environmental Engineering, Cornell University, Ithaca, New York, USA; c Department of Civil and Environmental Engineering, McCormick School of Engineering and Applied Science, Northwestern University, Evanston, Illinois, USA; Shanghai Jiao Tong University

**Keywords:** ecophysiology, *Paraburkholderia*, nominal oxidation state of carbon, time-resolved metabolic footprinting, carbon use efficiency

## Abstract

We used time-resolved metabolic footprinting, an important technical approach used to monitor changes in extracellular compound concentrations during microbial growth, to study the order of substrate utilization (i.e., substrate preferences) and kinetics of a fast-growing soil isolate, *Paraburkholderia* sp. strain 1N. The growth of *Paraburkholderia* sp. 1N was monitored under aerobic conditions in a soil-extracted solubilized organic matter medium, representing a realistic diversity of available substrates and gradient of initial concentrations. We combined multiple analytical approaches to track over 150 compounds in the medium and complemented this with bulk carbon and nitrogen measurements, allowing estimates of carbon use efficiency throughout the growth curve. Targeted methods allowed the quantification of common low-molecular-weight substrates: glucose, 20 amino acids, and 9 organic acids. All targeted compounds were depleted from the medium, and depletion followed a sigmoidal curve where sufficient data were available. Substrates were utilized in at least three distinct temporal clusters as *Paraburkholderia* sp. 1N produced biomass at a cumulative carbon use efficiency of 0.43. The two substrates with highest initial concentrations, glucose and valine, exhibited longer usage windows, at higher biomass-normalized rates, and later in the growth curve. Contrary to hypotheses based on previous studies, we found no clear relationship between substrate nominal oxidation state of carbon (NOSC) or maximal growth rate and the order of substrate depletion. Under soil solution conditions, the growth of *Paraburkholderia* sp. 1N induced multiauxic substrate depletion patterns that could not be explained by the traditional paradigm of catabolite repression.

**IMPORTANCE** Exometabolomic footprinting methods have the capability to provide time-resolved observations of the uptake and release of hundreds of compounds during microbial growth. Of particular interest is microbial phenotyping under environmentally relevant soil conditions, consisting of relatively low concentrations and modeling pulse input events. Here, we show that growth of a bacterial soil isolate, *Paraburkholderia* sp. 1N, on a dilute soil extract resulted in a multiauxic metabolic response, characterized by discrete temporal clusters of substrate depletion and metabolite production. Our data did not support the hypothesis that compounds with lower energy content are used preferentially, as each cluster contained compounds with a range of nominal oxidation states of carbon. These new findings with *Paraburkholderia* sp. 1N, which belongs to a metabolically diverse genus, provide insights on ecological strategies employed by aerobic heterotrophs competing for low-molecular-weight substrates in soil solution.

## Abstract (precis)

Exometabolomic footprinting methods have the capability to provide time-resolved observations of the uptake and release of hundreds of compounds during microbial growth. Of particular interest is microbial phenotyping under environmentally relevant soil conditions, consisting of relatively low concentrations and modeling pulse input events. Here, we show that growth of a bacterial soil isolate, *Paraburkholderia* sp. 1N, on a dilute soil extract resulted in a multiauxic metabolic response, characterized by discrete temporal clusters of substrate depletion and metabolite production. Our data did not support the hypothesis that compounds with lower energy content are used preferentially, as each cluster contained compounds with a range of nominal oxidation states of carbon. These new findings with *Paraburkholderia* sp. 1N, which belongs to a metabolically diverse genus, provide insights on ecological strategies employed by aerobic heterotrophs competing for low-molecular-weight substrates in soil solution.

## INTRODUCTION

Low-molecular-weight (LMW) compounds in soil solution comprise a small amount of total soil carbon yet represent an important carbon pool at the interface between the decomposition of larger residues and subsequent assimilation by the soil microbial community (1). The LMW C pool is typically less than 10% of soil solution C and totals to 1 to 2 g of C m^−2^ down to a meter depth in typical forest soils (2). Because of their small compound size (<1 kDa) and the presence of specific and nonspecific membrane transport systems in microbial cells, LMW compounds are rapidly assimilated and mineralized by the microbial community (3). The composition of the LMW fraction of soil solution reflects common precursors and intermediates found in the metabolism of plant and microbial cells (4). These compounds, primarily sugars, amino acids, and organic acids, are maintained at low concentrations by a balance of microbial uptake and a constant supply from metabolite release, cell death, root exudation, depolymerization, and downward movement of soil solution (5). Estimates of half-lives of specific compounds within this class vary from less than an hour to more than 7 days depending on the soil horizon being investigated (6–8). Though rapidly cycled, these compounds are thought to disproportionately make up persistent C and N pools after transformation (1) and are increasingly being represented in finer resolution in microbially explicit models to predict stabilization rates (9).

Compound uptake preferences and metabolic use efficiency (carbon use efficiency [CUE], synonymous with microbial growth yield) of LMW C and N is predetermined and regulated by the individual microbial cell (10) as well as influenced by environmental stoichiometric limitations (11) and the presence and availability of other organic substrates (12). A current question is whether we may determine first principles for the diverse metabolic potential inherent in the soil microbial community. Previous exometabolomic trials with bacteria and yeasts have shown almost no links between genetic or ecotype relatedness and substrate uptake patterns (13) to strong genotypic clustering or mixed clustering in relation to substrate uptake (14, 15). There is evidence for (16) and against (17) whether individual bacterial populations exhibit conserved uptake preferences when diverse resources are abundant. Quantitative stable isotope probing work on complex, *in situ* soil communities suggests that evolutionary background may be the main constraint on carbon assimilation (18). One relatively well-defined ecophysiological paradigm that appears to hold true for soil bacteria is the broad distinction between copiotrophic (*r*-selected) and oligotrophic (*K*-selected) life histories (19). In this framework, copiotroph populations have higher growth rates, are adapted to pulsed input events, and tend to exhibit inefficient conversion of LMW C into biomass (10). Our current capacity to model LMW C cycling in soil solution may be limited by our ability to define the predominant ecophysiological characteristics that govern heterotroph activity.

There is clear evidence from LMW tracer experiments in the field (20–22) and laboratory incubations (23–25) that oxidized compounds are taken up more rapidly by the microbial community, followed by a less efficient conversion of that C to biomass (lower CUE) in surficial soil horizons. These observations are often associated with an inferred relationship between the energetic content of a carbon substrate (12, 26) and its potential CUE. Generally, a compound with a higher nominal oxidation state (NOSC) will require more energy, in the form of reducing equivalents, to reduce the carbon to the oxidation state of the biomass being created (∼−0.2). In other words, growth on substrates with a NOSC above that of biomass (27) will reduce growth efficiency due to energy limitations, while substrates with a NOSC far below that of biomass will be carbon limited during growth (28). It is also possible that under mixed-substrate utilization, the more oxidized compound could be used primarily for energy generation alone (12). This may explain earlier and less efficient use of higher-NOSC compounds in these studies. It has been hypothesized that the relationship between NOSC and CUE may resemble a bell-shaped curve centered around the NOSC of biomass (29). However, under aerobic conditions, a more likely relationship is a linear increase to a theoretical maximum with increasing energetic content (lower NOSC) (30).

Relationships between compound identity, uptake, and CUE are not as clear at the microbial population level. Substrate preferences can be extremely divergent, even across similar fast-growing isolates (31). A traditional view is that these fast-growing organisms, which are considered copiotrophs, should stagger uptake based on potential maximal growth rate on that substrate (19). This phenomenon, known as catabolite repression, is the classic paradigm of microbial substrate selection and often exhibits diauxic growth patterns (32, 33).

Much of the theoretical underpinning for the hypotheses that higher-NOSC compounds are more readily assimilated or those that support catabolite repression are based on simplified, minimal media (34). The NOSC framework, though field tested, is built upon single-carbon-source-culturing trials (30), and deviations can be observed under even simple mixed-substrate-culturing conditions (12). There is also an assortment of observations showing simultaneous use of substrates that provide medium or low maximum growth rates when low-concentration, mixed-substrate media are used (35). It is imperative that appropriate media mimicking the relevant ecological niches be used to develop relationships between compound characteristics and C utilization (36). Time-resolved metabolic footprinting provides an avenue for addressing these concerns (17). This approach uses sigmoidal model fits to define the midpoint of substrate depletion from the media (*t*_50_) and parse out substrate preferences during microbial growth. It has been used in conjunction with both natural (37–39) and defined, complex media (40) and has shown the ability to link isolate exometabolite profiling with *in situ* community function (41).

In this work, we utilized time-resolved exometabolomic footprinting to profile substrate preferences and CUE of the fast-growing soil bacterium *Paraburkholderia* sp. strain 1N (42). *Paraburkholderia* is a relatively newly defined genus created from the subdivision of *Burkholderia* and predominantly isolated from bulk soil and the rhizosphere or found as endophytes (43–45). This organism has been found to preferentially respond to additions of phenolics and drive priming in forested soils (46). *Paraburkholderia* sp. 1N was isolated using an undefined, complex growth medium referred to as soil-extracted solubilized organic matter (SESOM) (47) derived from an organic rich surface horizon in a hemlock-hardwood stand. SESOM was used to recreate conditions encountered in soil solution following rainwater infiltration and residence in a surficial forest soil when fast-growing populations are most active. The depletion of naturally occurring organic substrates as well as appearance of metabolites was monitored in batch study using liquid chromatography (LC) coupled with high-resolution mass spectrometry (HRMS) as well as ^1^H nuclear magnetic resonance (NMR) analyses. Our aim was to phenotype this fast-growing soil microorganism under realistic, pulsed input conditions. We tested for relationships between substrate chemical parameters (NOSC, initial concentration) and microbial preferences (*t*_50_) and depletion rates, as well as explored potential links between the aforementioned parameters and CUE.

## RESULTS

### Isolation and genomic characterization.

The forest soil isolate *Paraburkholderia* sp. 1N was isolated on soil-extracted solubilized organic matter (SESOM) and classified to the genus *Paraburkholderia* (see Fig. S2 in the supplemental material) (42). Draft genome size was measured to be 11.1 Mb, containing 353 contigs, with a GC content of 60.6%. The annotated draft genome was found to contain 11,964 coding genes with 4,045 distinct functions. Only 3,704 genes could be matched with SEED annotation ontology. The largest portions of annotated genes are assigned functions that fall within carbohydrate metabolism (*n* = 1,039), amino acid metabolism (*n* = 666), and metabolism of aromatic compounds (*n* = 487) (Fig. S3). The phosphoenolpyruvate protein of the phosphotransferase system (PTS), a type of phosphotransferase system implicated in well-studied carbon catabolite repression systems, is present (EC 2.7.3.9). Annotated ATP-binding cassette (ABC) transporters of interest include the presence of the branched-chain amino acid uptake systems (Liv, TC 3.A.1.4.1), an oligopeptide uptake system (Opp, TC 3.A.1.5.1), and a dipeptide system (Dpp, TC 3.A.1.5.2). Further characterization of this isolate can be found in the work of Wilhelm et al. (42).

### SESOM provides a complex, realistic growth medium.

SESOM was created from the organic (Oa) horizon of the hemlock-dominated stand, and initial characterization was conducted using a range of analytical techniques. SESOM had a yellowish hue and was acidic (Table 1). Potassium was the major cationic component, with appreciable amounts of sodium, aluminum, and iron as well (Table S1). There was no detectable, dissolved inorganic carbon at this pH, and therefore, the entirety of measured solution carbon was organic. The solution had an observed C/N ratio of 16.4. Approximately 16% of solution nitrogen (TN) was in the form of NH_4_^+^, while 41% was found to be in the ninhydrin-reactive pool and was considered to be predominantly in the form of amino acids. The remaining 43% was uncharacterized but organic in nature. Reducing sugars accounted for 16.5% of total organic carbon (TOC) in the solution. Specific LMW compounds of interest were quantified using two different targeted LC-HRMS and ^1^H NMR measurements (Table 2; see also Materials and Methods). In total, compounds targeted using LC-HRMS and ^1^H NMR and colorimetric methods accounted for 19.5% of TOC and 39.9% of TN. Many additional compounds could be putatively identified using untargeted approaches but could not be quantified. Glucose is the dominant sugar present and the only sugar estimate that could be reliably made from ^1^H NMR spectra (Table 2 and Fig. S1). Valine, alanine, and glutamate were the dominant amino acids present, while acetate, lactate, and gluconate were the predominant organic acids present out of those quantified (Table 2).

**TABLE 1:** SESOM initial solution characteristics

Component	Value	Unit
pH	3.55	
EC	164	μS/cm
		
TOC	183.1	mg of C/liter
TN	11.3	mg of N/liter
NH_4_^+^	1.79	mg of N/liter
Ninhydrin-N	4.59	mg of N/liter
Reducing sugars, C	30.7	mg of C/liter

**TABLE 2:** Targeted substrate initial concentrations and depletion dynamics

Compound	Symbol	Units	Source^*a*^	Type^*b*^	Initial concn	*a*	*o*	*t*_50_	*w*	%^*c*^	NOSC
Pyruvate	pyr	μM	M	1	0.37	0.37	0.00			0.03	0.67
α-Ketoglutarate	akg	μM	M	1	0.59	0.43	0.16			0.05	0.80
Ornithine	orn	μM	A	1	0.96	0.43	0.53			0.05	−0.40
Methionine	met	μM	A	1	1.03	0.75	0.28			0.09	−0.40
Phenylalanine	phe	μM	A	1	1.08	0.57	0.51			0.12	−0.33
Malate	mal	μM	M	1	1.21	0.93	0.28			0.09	1.00
Histidine	his	μM	A	1	1.65	1.27	0.37			0.18	0.67
Lysine	lys	μM	A	1	1.67	1.19	0.48			0.17	−0.67
Citrulline	cit	μM	A	1	2.24	1.57	0.67			0.36	−0.80
Arginine	arg	μM	A	1	3.00	2.70	0.31			0.38	0.33
2-Keto-d-gluconate	2kg	μM	M	1	3.20	3.04	0.16			0.42	0.67
Asparagine	asn	μM	A	1	4.24	4.24	0.00			0.39	1.00
Serine	ser	μM	A	1	6.00	6.00	0.00			0.42	0.67
Succinate	succ	μM	M	1	6.57	6.57	0.00			0.61	0.50
Glutamine	gln	μM	A	1	9.05	8.53	0.71			0.97	0.40
Aspartate	asp	μM	A	1	11.50	11.50	0.00			1.07	0.75
Gluconate	glucon	μM	M	1	17.63	17.26	0.37			2.37	0.33
Glutamate	glu	μM	A	1	54.92	54.92	0.00			6.23	0.40
Alanine	ala	μM	H	1	70.00	44.00	26.00			3.06	0.00
Acetate	ace	μM	H	1	95.60	72.53	23.07			3.37	0.00
Proline	pro	μM	A	2	0.83	0.43	0.40	16.29	0.57	0.05	−0.40
Isoleucine	ile	μM	M	2	1.94	1.92	0.02	16.79	0.99	0.27	−1.00
Leucine	leu	μM	M	2	9.55	9.51	0.04	16.93	0.52	1.33	−1.00
Tryptophan	trp	μM	A	2	0.62	0.62	0.00	17.06	0.19	0.16	−0.18
Tyrosine	tyr	μM	A	2	0.65	0.65	0.00	17.08	0.08	0.14	−0.22
Citrate	citr	μM	M	2	2.59	2.55	0.04	17.33	0.36	0.18	1.00
Lactate	lac	μM	H	2	24.94	7.34	17.60	17.90	1.09	0.65	0.00
Threonine	thr	μM	A	2	6.40	5.91	0.49	17.93	0.62	0.55	0.00
Ammonium	NH_4_^+^	mg N/liter	C	2	2.07	2.07	0.00	20.32	0.78		
Reducing sugars^*d*^		μM	C	2	430.42	248.35	182.07	20.42	1.47	31.29	0.00
Valine	val	μM	A	2	168.29	168.29	0.00	20.52	0.72	19.96	−0.80
Glucose	glc	μM	H	2	192.01	134.82	57.19	21.06	1.15	17.82	0.00
Unidentified	Unknown aromatic	AUC	H	2	1.00	0.62	0.38	21.82	0.14		

a “Source” refers to the analytical technique used (M, LC-HRMS-Metabo method; A, LC-HRMS-AA method; H, ^1^H NMR; C, colorimetric).

b Type 1 refers to substrates depleted before the first sampling point, while type 2 substrates have been fit to a sigmoidal depletion curve: $y=\frac{a}{1+e^{\frac{x-t_{50}}{w}}}+o$

c Refers to percentage of total C assimilated (51.8 mg of C/liter) that could be attributable to the targeted substrate.

d Reducing sugars method includes glucose values.

**FIG 1:**
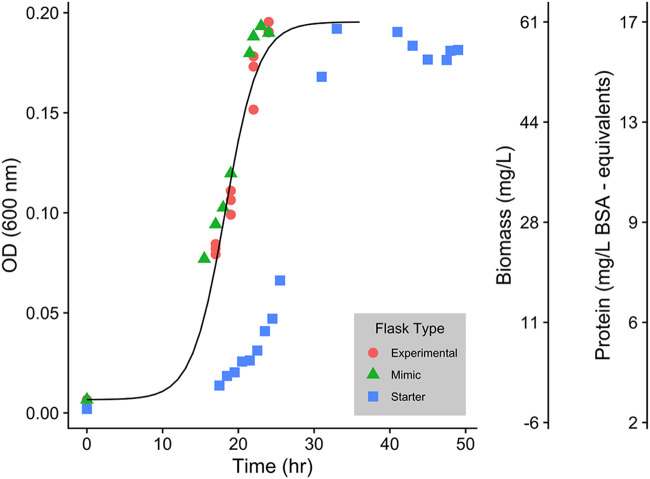
Growth curve of *Paraburkholderia* sp. 1N on SESOM. Data were collected from experimental replicates (*n* = 3), a mimic flask (*n* = 1), and a starter flask (*n* = 1) used in the experiment. The *y* axes display optical density measured photometrically at 600 nm as well as a biomass conversion (biomass [milligrams per liter] = 343.09 OD_600_ – 5.38) and cellular protein content (protein [milligrams per liter BSA equivalents] = 67.19 OD_600_ + 2.78). Biomass was measured only on experimental replicates via 0.2-μm filtration and protein content using a Bradford assay after cell lysis using dual analytical replicates. The starter flask was used for initial inoculation (OD_600_ = 0.0658) and is pictured to provide support for forcing the model fit.

**FIG 2:**
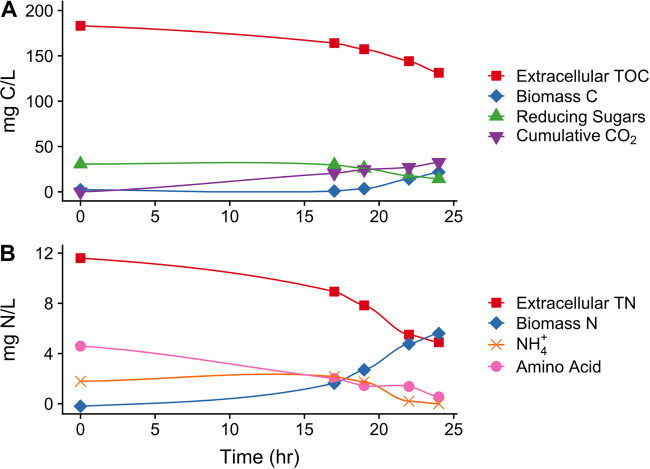
Carbon and nitrogen dynamics during *Paraburkholderia* sp. 1N growth on SESOM. Panel A displays changes to carbon pools, and panel B displays nitrogen pools. All points represent means (*n* = 3) with standard error bars (smaller than point size in all cases).

*Paraburkholderia* sp. 1N was grown aerobically in SESOM and growth rate (μ_max_) was determined to be 0.17 h^−1^ (generation time = 4.1 h) using linear fitting during exponential growth (Fig. 1). During the course of growth, 51.8 mg of C/liter (28.3% of initial TOC) was assimilated and 32.5 mg of C/liter (17.7% of initial TOC and 62.8% of assimilated C) was lost from the culturing vessel entirely (Fig. 2). This amount was assumed to be predominantly respiratory CO_2_ losses from the system (Fig. 2A, cumulative CO_2_), though it could have contained small amounts of volatile organic carbon as well. Biomass production reached a mean plateau of 21.7 mg of C/liter at stationary phase. Reducing sugars could potentially explain a large portion of C assimilated from SESOM medium (up to 31% of total C assimilated [Table 2]). Similarly, nitrogen was assimilated from SESOM medium during growth (Fig. 2B). Initially, amino acid N concentrations decreased, followed by a switch to predominantly NH_4_^+^ before a return to amino acid N depletion as NH_4_^+^ concentrations dropped below detection (Fig. 2B). Approximately 6.7 mg of N/liter was removed from solution and incorporated in the biomass of *Paraburkholderia* sp. 1N. Total amino acid N (4.05 mg of N/liter) and total NH_4_^+^ N depletion (1.79 mg of N/liter) accounted for all but 0.86 mg of N/liter removed from solution, indicating a smaller contribution of alternative N sources to microbial uptake (Fig. 2B). There was no significant loss of N from the system.

**FIG 3:**
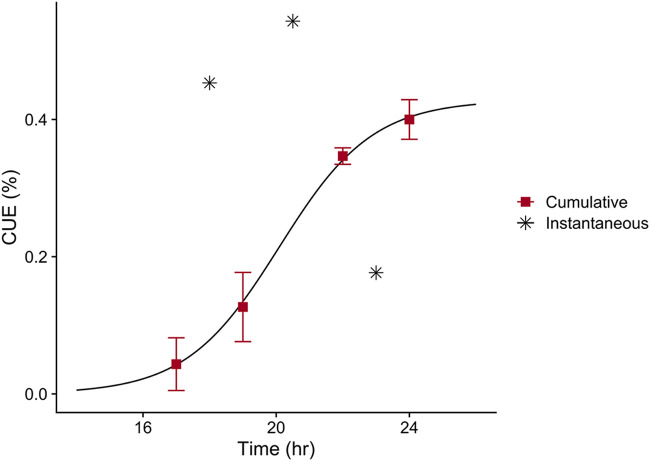
Carbon use efficiency (CUE) during microbial growth of *Paraburkholderia* sp. 1N on SESOM. All points represent means with standard error bars (*n* = 3). Instantaneous CUE was calculated from growth in between biomass sampling points, so estimated values are displayed at the center of the measurement period. A similar 4-point sigmoidal curve was fit for cumulative CUE, $y=\frac{a}{1+e^{\frac{-(x-t_{50})}{w}}}+o$, where *a* = 0.43, t*_50_* = 20.09, *w* = 1.40, and *o* = 0.

Estimates of cumulative CUE show an increase through the growth curve to a plateau of 0.43 (Fig. 3). Instantaneous estimates were calculated over each measurement period to determine if higher instantaneous rates were achieved. A maximum of 0.54 was estimated over the 19- to 22-h portion of the experiment, which encompasses the inflection point of the growth curve. Instantaneous CUE rapidly declined after the inflection point as growth slowed and the population entered stationary phase.

### Time-resolved exometabolomic footprinting provides information on compound depletion and appearance patterns.

**FIG 4:**
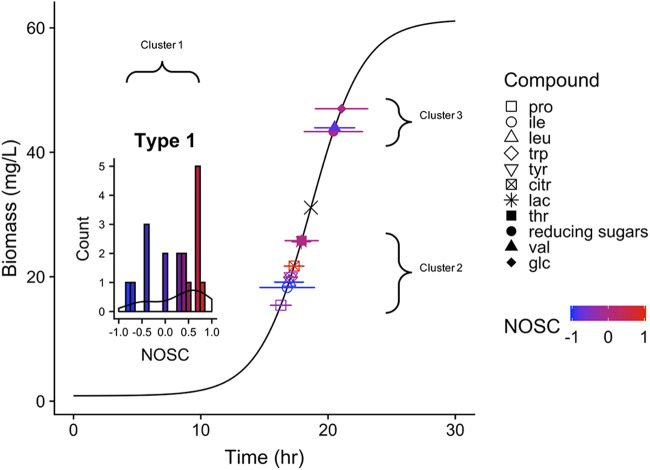
Usage window plot of *Paraburkholderia* sp. 1N growth on SESOM. Modeled depletion patterns of targeted carbon substrates are overlaid on the growth curve. Only substrates for which sufficient data allowed the fitting of a sigmoidal curve are depicted in the plot (type 2). The points represent the inflection point of depletion (*t*_50_), and the horizontal bars represent the 90% usage window (modification of the fit window, *w*). The inflection point of the growth curve is also overlaid on the figure (×). Many substrates were depleted substantially before the first sampling point (type 1 [Fig. S3]), and therefore, no kinetic data are available. A histogram of the targeted type 1 substrates is shown in the early portion of the growth curve for this reason. All depicted substrates are colored by their oxidation state (NOSC) and listed in order of increasing *t*_50_ in the key. Lactate is mostly obscured by threonine, which has a slightly later *t*_50_.

LC-HRMS and ^1^H NMR were used for the direct quantification of 31 targeted compounds at each time point sampled during the growth of *Paraburkholderia* sp. 1N in SESOM (Table 2). Depletion patterns were analyzed by overlaying the *t*_50_ and 90% usage window of each substrate onto the growth curve of the organism (Fig. 4) (17, 31). Concentrations of all of the 31 targeted compounds decreased during the course of growth. It is important to note that the technique does not independently confirm the presence of these compounds intracellularly, and the terms depletion and usage are employed throughout this paper instead of uptake and assimilation. There is a possibility of extracellular transformation without uptake or even uptake without subsequent usage (38).

Substrate depletion from the media occurred in three distinct clusters, as determined by usage window plotting (Fig. 4) (17). The first cluster, containing 20 of the 31 substrates, occurred predominantly before the first sampling event and the substrate depletion pattern was either entirely or almost entirely missed, making them type 1 substrates (Fig. 4, inset, and Fig. S4; see Materials and Methods). The first cluster of compounds consisted of a mixture organic acids and amino acids (Table 2 and Fig. 4). There was a slightly higher abundance of compounds with NOSC greater than 0 in this earlier phase of depletion (Fig. 4, inset). The remaining 11 substrates were removed from the media during the sampling period and could be fit to a sigmoidal uptake curve (type 2 substrates [Fig. S5]). Type 2 substrates fell into two separate clusters of overlapping usage, one before the inflection point of growth and one occurring afterwards (Fig. 4). The second cluster contained mainly amino acids (proline, isoleucine, leucine, tryptophan, and tyrosine) as well as two organic acids (citrate and lactate) (Table 2 and Fig. S5). The third cluster consisted of glucose and valine disappearance as well as the removal of unidentified aromatic peaks (identified via ^1^H NMR and assumed to be attributed to tannin-like molecules) coupled with NH_4_^+^ uptake (Fig. 4 and Fig. S5). Total reducing sugars had an earlier *t*_50_, indicating that the multiple other, lower-concentration, sugars had a slightly earlier removal. This later cohort of compounds comprise the largest concentrations of available C and N in solution out of the substrates quantified. Targeted substrates, overall, represented 74.9% and 74.4% of total C and N depletion, respectively (Table 2).

**FIG 5:**
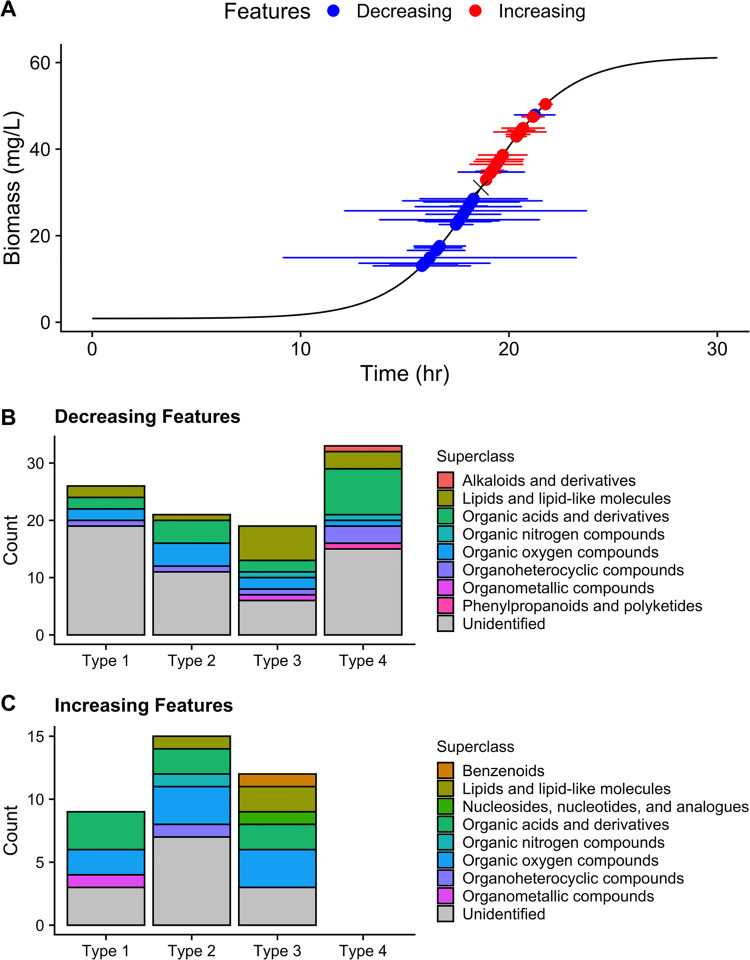
Untargeted features detected split by directionality of change. Type 2 features whose depletion or appearance could be modeled using a sigmoidal fit are depicted in a usage window plot (A). Points are overlaid over the growth curve at the inflection point of their depletion or appearance (*t*_50_), while the horizontal bars represent the 90% usage window (modification of the fit window, width) of the feature. Decreasing and increasing features were sorted based on the kinetics and shape of their curve and sorted by superclass (B and C; type 1, early depletion or appearance/insufficient data for fit; type 2, sigmoidal curve fit; type 3, nonsigmoidal depletion or appearance; type 4, late depletion or appearance/insufficient data for fit).

An untargeted approach was then employed to look for compounds beyond those targeted previously. In total, 135 significant features were detected and the area under the curve was quantified. Of the 135 detected features, 99 decreased and 36 increased during the growth of *Paraburkholderia* sp. 1N (Fig. S6 to S12). Only 21 of the decreasing features could be fit using equation 2 in Materials and Methods (Fig. S7), and only 15 of increasing features could be fit with the modified version of the same equation (Fig. S11). A combined plot was used to visualize the depletion and appearance of these features (Fig. 5A). There was a clear distinction between the *t*_50_ of decreasing and increasing features, with the mean *t*_50_ of decreasing features occurring 2.4 h before that of the increasing features [two-sample *t*(34) = −6.3742; *P* < 0.001]. Compound appearance tended to occur over smaller intervals (90% usage window) than substrate depletion, with a 3.3-h-longer usage window estimate for decreasing features [Wilcoxon rank sum W(34) = 279; *P* < 0.001].

Inclusion of untargeted features show that *Paraburkholderia* sp. 1N exhibited metabolic diversity in the variety of compounds that could be depleted from SESOM media. Similar levels of superclass diversity were removed from the extracellular matrix as were transformed or released as metabolites or upon cell death (*n* = 8) (Fig. 5B and C). Alkaloids and phenylpropanoids and polyketides were only removed from SESOM media, and both superclasses showed late disappearance (Fig. 5B, type 4). Benzenoids and nucleosides, nucleotides, and analogues were only released into the SESOM media, and both superclass categories of compounds showed irregular appearance profiles (Fig. 5C, type 3). Of the untargeted features that could be fit to a sigmoidal usage pattern (Fig. 5B and C, type 2), similar ranges of superclasses were involved (lipids, organic acids, organic nitrogen compounds, organic oxygen compounds, and organoheterocyclic compounds).

### Relationships between substrate depletion and hypothesized predictor variables (NOSC, specific μ_max_, and concentration).

**FIG 6:**
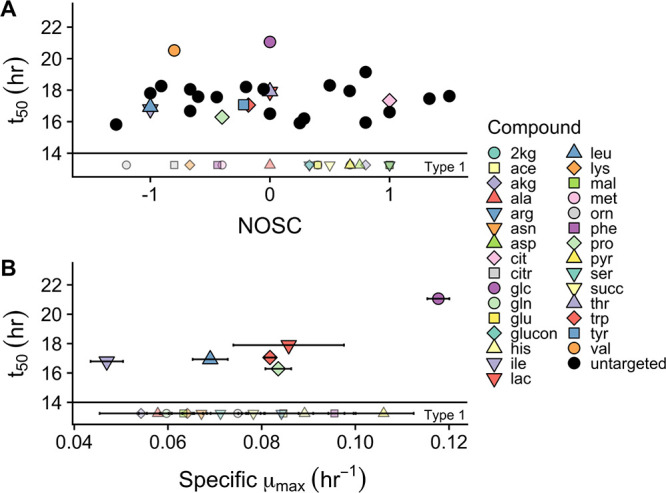
Model fit *t*_50_ values for substrates as a function of hypothesized predictor variables. (A) The midpoint of depletion, t_50_, as a function of substrate nominal oxidation state of carbon. (B) *t*_50_ as a function of the specific growth rate (μ_max_) of *Paraburkholderia* sp. 1N growing on that substrate as the sole C source. Error bars represent standard errors (*n* = 3). Both panels share the same key, though untargeted features are displayed only in Panel A. For both panels, type 1 substrates are depicted below the horizontal line and grayed out, as they have an *x* axis value (NOSC, specific μ_max_) but could not be fit to a 4-point sigmoidal depletion curve.

**FIG 7:**
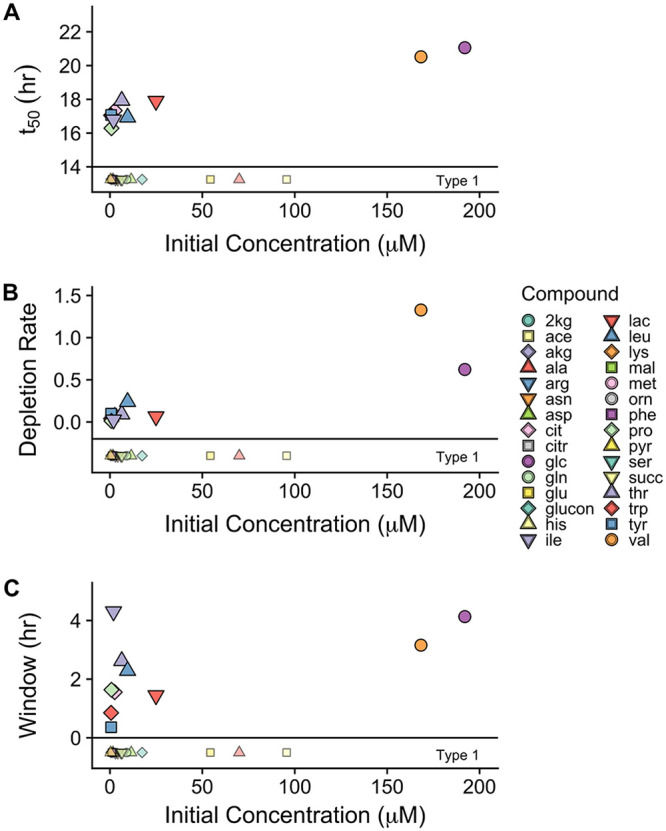
Model fit *t_50_* values for substrates (A), depletion rate (B), and usage window (C) as a function of initial concentration. Depletion rate is depicted normalized to biomass (millimoles per hour per gram of cells [dry weight]). The window depicted (Panel C) is the estimated 90% usage window. Type 1 substrates are depicted below the horizontal line and grayed, as they have *x* axis value (initial concentration) but could not be fit to a 4-point sigmoidal depletion curve.

We did not find NOSC or the sole C source growth rates, specific μ_max_, to be reliable predictors of preferential substrate depletion, *t*_50_ (Fig. 6). The inclusion of untargeted, putatively characterized NOSC values (Fig. 6A) did not help build any relationship between the two variables. Specific μ_max_ was determined by growing *Paraburkholderia* sp. 1N on each substrate individually to determine maximal potential growth. While there was an upward trend between maximal growth rate (specific μ_max_) and *t*_50_, it is clear that inclusion of any of the type 1 substrates would invalidate that relationship (Fig. 6B). Similarly, there appears to have been an upward trend between initial compound concentration and substrate depletion preferences (*t*_50_ [Fig. 7A]), yet inclusion of three of the type 1 substrates, acetate, alanine, and glutamate, is not possible in such a relationship. Compounds at higher initial concentrations appear to have been used at higher rates (Fig. 7B) and for a longer window (Fig. 7C). Inclusion of type 1 substrates could maintain a nonlinear relationship, yet at this time only two points, glucose and valine, were driving the trend. More data from earlier in the growth curve would be necessary to confirm these relationships.

## DISCUSSION

Three broad clusters of substrate depletion could be outlined during growth (Fig. 3). Many substrates belonging to the first cluster were depleted to their maximum potential before subsequent depletion of the second cluster began (Table 2 and Fig. S4). Distinct clustering of time-dependent substrate depletion implies multiauxic growth behavior of *Paraburkholderia* sp. 1N on SESOM. Within each phase of substrate use, there was coutilization of multiple substrates, which is similar to observations for other fast-growing *Bacillus* and *Pseudomonas* species (17, 31). The coexistence of sequential (multiauxic) and simultaneous (coutilization) substrate use as a metabolic strategy is more common than previously thought from investigation of the growth of heterotrophs in diverse, low-concentration media (48, 49). We observed that acetate, alanine, and glutamate likely provided the majority of carbon from SESOM for *Paraburkholderia* sp. 1N’s biomass production in the first phase, lactate in the second, and reducing sugars coupled with valine in the final phase (Table 2 and Fig. 6). Inclusion of untargeted data indicates that there may have been many more compounds being used over longer periods within this multiphasic substrate removal pattern (Fig. 4A). Unfortunately, the inability to quantify putatively identified compounds leaves their potential contribution to biomass production unknown for this experiment.

In complex media, such as SESOM, translation and expression of all transporter systems and metabolic pathways present too high a cost (50), requiring a complex regulatory network to shift uptake dynamics. The observed clustering of substrate depletion is indicative of a metabolic network shift, though higher-resolution growth measurements are needed to determine whether *Paraburkholderia* sp. 1N is able to make these types of metabolic shifts with or without a slowdown in growth rate (51, 52). *Paraburkholderia* sp. 1N has a putative phosphoenolpyruvate protein of the phosphotransferase system (PTS), which is implicated in carbon catabolite repression in Escherichia coli as well as Bacillus subtilis and the selective use of sugars (33). Though a PTS system is present, there was no significant difference in the mean uptake of reducing sugars and glucose to suggest carbon catabolite repression. *Paraburkholderia* sp. 1N also has the Liv (leucine-isoleucine-valine) system, which is an ATP-binding cassette (ABC) transporter found in E. coli (53). It may be possible that *Paraburkholderia* sp. 1N can up- and downregulate one of the six annotated, amino acid-binding proteins (TC 3.A.1.4.1). These have been shown to be paralogues in other organisms and differ in amino acid specificity and affinity (53). Up- and downregulation of transporters with different affinities could explain the observed clustering of amino acid uptake patterns. Alternatively, similar ABC transporter systems have been found to have broad specificity (54), so another possibility is that the uptake of amino acids may not be under strict control at all. The predominance of increasing features in the exometabolome after the inflection point of growth (Fig. 5A), at timing similar to that of the switch to multiple sugar and valine metabolism, is indicative of a regulated metabolic shift. This may be due to changing nutrient availability or other stress encountered during batch growth and a resulting release of metabolites (55).

*Paraburkholderia* sp. 1N exhibits a growth strategy that falls outside traditional paradigms. While this isolate did show metabolic diversity that is in accordance with that of other fast-growing soil bacteria, it did not selectively use those substrates (glucose, histidine, phenylalanine, pyruvate, and lactate) that connote optimal growth rates (Fig. 7B). Coutilization of carbon sources is expected to boost microbial growth rate (33, 56), though at the expense of individual substrate uptake rates (57). While it is possible that clustered substrate usage may offer the advantage of an increased growth rate in a way not observed in individual specific μ_max_ estimates (Fig. 5B), this seems unlikely, as substantially lower biomass normalized depletion rates (millimoles per hour per gram of cells [dry weight]) were observed earlier during growth (Fig. 6B). The lower depletion rates of earlier type 2 substrates may indicate a strategy of maximizing growth efficiency using these lower-concentration substrates even though they do not support higher growth rates. Either *Paraburkholderia* sp. 1N does not group with other copiotrophs or not all copiotrophs maximize growth rate at the expense of efficiency (10). Other observations of the coexistence of multiauxic and simultaneous substrate use tend to reinforce the idea that substrate selection is still based on affording the highest growth rate possible (31, 49). Bacillus cereus is one other example of a fast-growing, *r*-selected organism that has been observed to selectively take up substrates based on a mechanism outside maximizing growth rate (31).

Our results did not show that *Paraburkholderia* sp. 1N had any predilection for depleting more oxidized substrates first, contrary to observations from whole-soil communities (24). There was a slightly higher number of oxidized type 1 substrates (Fig. 3, inset), yet overall, there was no relationship between these parameters (Fig. 5). The form of regulation that best fits our observations is the multiauxic usage of substrate groups representing a mix of divergent oxidized and reduced compounds. This use of a more oxidized compound for energy generation, resulting in increased growth yield, has been frequently observed in simple, two-substrate mixtures (12). Increasingly, the coutilization of substrates is thought to be the result of optimal enzyme allocation (58), suggesting an advantage to the simultaneous use of glycolytic and gluconeogenic substrates (59, 60). Indeed, recent ^13^C labeling experiments with a marine heterotroph have shown the “different and complementary roles” of simultaneously used amino acids (49). There is some evidence for this in the second grouping of substrates in which growth on citrate (NOSC = 1.00) was coupled with isoleucine and leucine (NOSC = −1.00), among others. This pattern of C utilization was observed in our data because of our comprehensive tracking of multiple metabolites through time and grouping by metabolite use stage. This phenomenon may be missed if the reduced compound in the coupled pair or group is not also tracked, which is a possibility in many whole-soil data sets probing a small subset of isotopically labeled substrates (20, 22, 24).

The efficiency of biomass production of *Paraburkholderia* sp. 1N (CUE) was observed to be within reported ranges of *in situ* soil communities and soil isolates. The estimated CUE of 0.43 is well within those reported for other bacterial pure cultures (CUE_P_, 0.2 to 0.8) as well as those reported for *in situ* soil communities (CUE_E_, 0 to 0.85) (28, 61). This value is close to those reported for chemostat cultures of Klebsiella aerogenes NCTC 418 (∼0.4) growing on limited glycerol concentrations in the <20 μM range, similar to many of the substrate concentration ranges in this medium (10). *Paraburkholderia* sp. 1N’s cumulative CUE also aligns well with the proposed relationship between maximal growth rate and carbon use efficiency and puts its CUE alongside that of a considered oligotroph, *Rhodospirillaceae* sp. PX3.14 (μ_max_ = 0.126 to 0.144; CUE ∼ 0.38) (62, 63). As mentioned previously, this high CUE and slow maximal growth rate, compared to those of other bacteria, may indicate that *Paraburkholderia* sp. 1N is on the boundary between the copiotrophic and oligotrophic ecological distinctions (62). Interestingly, predictions of CUE based on genome size alone show that *Paraburkholderia* sp. 1N’s estimated CUE of 0.43 is around the potential CUE of 0.4 predicted for its genome size of 11.1 Mb (64). Thus, while this organism may require greater maintenance resource allocation than others with smaller genomes, it is able to grow at an efficiency near its predicted potential in a highly diverse and carbon-limiting medium.

Predicting the use efficiency of individual substrates (65), based on temporal alignment between uptake and overall CUE, is a tempting next step but one that has many potential pitfalls. For instance, the second cluster of substrate utilization (Fig. 4) temporally aligns with the highest estimate of instantaneous CUE (Fig. 3). It might be reasonable to infer that this cluster of amino acids and organic acids is used most efficiently during this time of high biomass production, yet there is a possibility for a disconnect between assimilation and actual metabolic use. The population may employ a strategy of transforming the molecule to protect it from potential use by other microorganisms or for intracellular storage (66). The simultaneous assimilation of multiple substrates in distinct groups, which we observed (Fig. 3), could result in the microbial population preferentially routing one substrate to dissimilatory pathways and the other to assimilation (59). This would result in substrates with disparate individual use efficiencies that produce the observed average CUE.

Substrate concentration differences were up to 2 orders of magnitude in some cases for the SESOM used in this experiment (Table 2) and may have overwhelmed any influence of substrate energy content (NOSC) on utilization preference. Studies with equimolar initial substrate concentrations are needed for quantitation and identification of substrate uptake preferences. The application of isotopic substrate labeling in such studies could further probe hypothetical relationships between substrate groups and individual substrate use efficiencies. Understanding the effects of both compound identity and compound concentration is instrumental to the implementation of substrate uptake framework in environmental prediction models.

*Paraburkholderia* sp. 1N belongs to a clade of bacteria abundant in forest soils which broadly influence C cycling via phenolic acid-induced priming (46). We have shown that *Paraburkholderia* sp. 1N preferentially uses LMW substrates in SESOM in three distinct phases (Fig. 5). Lower-concentration amino acids and organic acids were used earlier on in the growth curve, followed by higher-concentration sugars and an amino acid coupled with NH_4_^+^ uptake (Fig. 5 and Fig. S5). Moving forward, time-resolved exometabolomic footprinting studies of key species in soil microbial communities of interest could aid our understanding of net observations of LMW cycling and the underlying mechanisms. To evaluate the physiological profile exhibited by *Paraburkholderia* sp. 1N as representative of other *r*-selected populations, further investigations of other species are needed, based on community dominance or ranging in phylogenetic or physiological differences.

## MATERIALS AND METHODS

### Preparation of SESOM as an undefined medium for isolation and growth.

Soil was collected under a hemlock-dominated stand in Arnot Forest near previous experimental plots (67, 68). Field-moist soil samples were immediately placed in a cooler and stored at 4°C until further processing (<24 h). Soil-extracted, solubilized organic matter (SESOM) was prepared using a modified water extraction procedure (47). Briefly, 40 g of air-dried Oa horizon from Arnot Forest was mixed with 200 ml of 18.2 MΩ-cm water in 250-ml Nalgene bottles and shaken on an end-to-end shaker for 1 h at room temperature. Bottles were then left to stand for 24 h and then sequentially filtered through 1.6-μm glass microfiber (GF/A), 0.45-μm polyethersulfone (PES), and then 0.2-μm PES filters to produce a filter-sterilized solution. Multiple bottles were extracted at once and combined to provide sufficient SESOM for experimental purposes. The extracted solution was diluted 2-fold for all growth experiments to produce concentrations that might be more realistically encountered in percolating pore water following a rainfall event.

### Isolation.

*Paraburkholderia* sp. 1N was isolated from field-moist B horizon found at the site. Briefly, agar plates (15 g/liter) were made with SESOM as the sole C source (10× dilution). For isolate enrichment, fresh soil was shaken with deionized (DI) water (1:10 ratio) for 1 h and then let sit for 24 h at room temperature. Serial dilutions were created using Winogradsky salts (69) and 100 μl was spread onto plates. Plates were incubated at room temperature in the dark for 3 to 14 days, and colonies were chosen at first appearance and restreaked on fresh SESOM 10×-dilution plates. Three separate plating rounds were conducted. Cellular morphology was determined microscopically, and growth was checked in liquid SESOM (2× dilution). *Paraburkholderia* sp. 1N has circular entire-colony morphology and is rod shaped. The isolate was stored on SESOM agar plates at 4°C, and single colonies were used to initiate a starter flask before each experiment of interest.

### DNA extraction, genomic analysis, and phylogenetic analysis.

Genomic DNA was extracted from pelleted cells from a liquid culture of the isolate on 10 ml of SESOM (2× dilution) (70) and submitted to the Cornell University Sequencing Facility for sequencing using three multiplexed runs of Illumina MiSeq Nano (2 × 250 bp). Raw sequencing data were quality preprocessed with Trimmomatic (v.0.32) (71) and FastX Toolkit (v.0.7) (72) and then assembled with SPAdes (v.3.10.1) (73). Open reading frames were predicted using Prodigal (v.2.6.2) (74). The assembly was then uploaded to the KBase web server (75) for further processing. A phylogenetic tree was constructed using the KBase application Insert Set of Genomes Into Species Tree (v.2.1.10), dependent on Fast-Tree2 (76) to provide context within 50 neighboring genomes. Genomic annotation was then conducted using the KBase application Annotate Microbial Assembly, which utilizes the RAST toolkit (77).

### Growth on SESOM.

Acid-washed and autoclaved 125-ml Erlenmeyer flasks were used for all growth curve experiments. A starter flask containing 50 ml of SESOM was inoculated with a single colony of *Paraburkholderia* sp. 1N and grown overnight on a shaker at 150 rpm until reaching log phase. A 0.5-ml subsample (optical density at 600 nm [OD_600_] = 0.0658, or ∼7.64 mg/liter of biomass) was used to inoculate each experimental flask for the growth curve assessment (starting biomass = 0.0757 mg/liter). Assessment of the growth curve was conducted using OD_600_. A growth curve was then fit using a 4-point sigmoidal function (Fig. 1).

To allow the tracking of substrate depletion during growth, three flasks were destructively harvested at four points along the growth curve (17, 19, 22, and 24 h). Points were chosen to cover the breadth of the exponential phase of growth and the beginning of stationary phase. While each flask was independent, they were treated as replicates for each time point in subsequent analyses. During each destructive-sampling event, 10 ml was removed for analyses on unfiltered components (TOC, TN, and cellular protein content) while the remaining 40 ml was filtered through 0.2-μm PES filters to remove cellular biomass and stored frozen in separate aliquots for further analyses (pH, TOC, TN, reducing sugars, NO_3_^−^, NO_2_^−^, NH_4_^−^, ninhydrin-N, ^1^H NMR, and LC-HRMS). Since all media were from a single extraction event and of limited volume, all initial medium values were analyzed only once for each analyte of interest. A Shimadzu TOC-V_CPN_ was used to measure nonpurgeable organic carbon (referred to as total organic carbon) and total nitrogen using a 2% acidification (0.2 M HCl) and 1.5 min sparge time using high-temperature (720°C) catalytic (Pt) oxidation. Cellular protein content was measured on dual analytical replicates using a modified Bradford protein assay using bovine serum albumin (BSA) as the standard (78). Reducing sugars were measured using a colorimetric alkaline ferricyanide reaction (79). The colorimetric Griess reaction method was used to measure NO_3_^−^ and NO_2_^−^ (80, 81). A modified Berthelot reaction was used to measure NH_4_^+^ (82). Total free amino acids were estimated using a ninhydrin method (referred to as ninhydrin-N) (83–85). All colorimetric samples were analyzed using a Shimadzu UV-2600 UV-visible (UV-Vis) spectrophotometer.

### Estimation of carbon use efficiency determined via cell filtration.

Estimates of cumulative CUE (milligrams of C in biomass per milligrams of C assimilated) could be derived from measurements of initial SESOM in comparison to sampled time points before and after cell removal via 0.2-μm filtration (PES),

(1) $$\text{CUE}=\frac{{\left(\text{unfiltered solution C}\right)}_{t}-(0.2\text{-}\mu \text{m-filtered solution C}{)}_{t}}{{\left(\text{unfiltered solution C}\right)}_{t-1}-{\left(\text{unfiltered solution C}\right)}_{t}}$$

where all values are in milligrams of C per liter. Carbon measurements were made using a Shimadzu TOC-V_CPN_ as described above. Due to the large size of the injection needle port, measurements of unfiltered solution contained microbial cells and resulting values represent cellular as well as extracellular carbon in solution.

### Time-resolved exometabolomic footprinting.

**(i) Targeted metabolites via ^1^H NMR.** Samples were analyzed by ^1^H NMR using modified methods previously reported for extracted soil solutions (47, 86). Briefly 30 ml of filtered SESOM medium was immediately frozen and lyophilized. Samples were then reconstituted with 300 μl of 18.2 MΩ-cm water. After vortexing, samples were buffered to pH 7.0 with an addition of 200 μl of sodium hydrogen phosphate (0.1 mM, pH 7.0) made with 25% D_2_O (vol/vol) to provide a lock signal and containing 1 mM sodium 3-trimethylsilyl-[2,2,3,3,-D_4_]-1-propionic acid (TMSP). Solutions were transferred to 5-mm NMR glass tubes (length, 7 in.; Wilmad “Economy”). Spectral referencing was conducted in reference to the TMSP (final concentration = 0.4 mM). All NMR spectra were collected at 500 MHz at room temperature on a Bruker AV 500 operated by Bruker TopSpin 3.5.7 using a 10% D_2_O and water peak suppression program (one-dimensional [1D] nuclear Overhauser effect spectroscopy [NOESY] with presaturation and spoil gradients [noesygppr1d]) with 32 scans/sample and a 5-s relaxation delay for a total of 256 transients. Within MestReNova (v.12.0.0-20080), spectra were Fourier transformed and zero-filled to 64,000 data points. Spectra were then linearly phase shifted and apodized using a 0-Hz exponential function. All spectra were manually phase corrected (PH0, −26; PH1, 6) and baseline corrected using the built-in polynomial fit function. All spectra were reference shifted so TMSP was 0.00 ppm. Lastly, residual water peak was removed using the signal suppression tool with selectivity at 24 centered on the 4.7-ppm signal. Compound identification was initiated by matching peaks of interest with suitable references (47, 87) as well as using online spectral data banks (Human Metabolome Database) to confirm multiplicity and chemical shift. Once identified, integrating regions were defined and used for integration on all samples (Table S2).

**(ii) Targeted metabolites via LC-HRMS.** Another set of filtered subsamples were immediately frozen at –20°C. These samples were thawed and analyzed using LC-HRMS. Samples were run on a Thermo Scientific Dionex Ultimate 3000 liquid chromatography system coupled to a Q Exactive orbitrap mass spectrometer. Two separate methods were employed: a reversed-phased approach using a Acquity ultraperformance liquid chromatography (UPLC) Waters C_18_ column (2.1 by 100 mm by 1.7 μm) as the stationary phase and negative electrospray ionization to identify metabolites (88, 89) (Table 2, LC-HRMS-Metabo method) as well as a hydrophilic interaction approach using a Waters XBridge column (4.6 by 100 mm by 3.5 μm) and electrospray ionization with polarity switching for amino acids (90) (Table 2, LC-HRMS-AA method). Quality control (QC) checks were run every 10 samples with a 30% standard-deviation (SD) limit. All data were analyzed using an internally constructed template within the Thermo Scientific Xcalibur 3.0 Quan browser. The template was built using standards of all identified compounds and run between 0 and 15 μM.

**(iii) Untargeted metabolites via LC-HRMS.** Data from the reversed-phase method were alternatively processed using an untargeted approach via XCMS online v.2.3.0 (91). Detailed processing parameters are included in the supplemental material. XCMS online output with CAMERA annotation was then imported and further processed using R 3.6.0 (92). Features of interest were further refined based on the following selection criteria: |ln(fold change)| > 1, *P* < 0.05, and maxint >10^7^). Metabolites already targeted were removed based on overlapping *m/z* (±0.001) and retention time (±30 s). The refined CAMERA output was then used as input for MetaboQuest (http://omicscraft.com/MetaboQuest/), where *m/z* was searched against several databases (PubChem, HMDB, LIPID MAPS, KEGG, MMCD, and METLIN) and the lowest error (parts per million) hit was chosen for putative identification (level 2) (93). Those returned results containing InChIKeys were then classified using ClassyFire (https://cfb.fiehnlab.ucdavis.edu/) (94). The superclass level, which includes 26 organic and 5 inorganic categories, was chosen as the most informative way to present compound differences as determined by this untargeted approach.

### Curve fits for substrate depletion.

Substrate depletion was modeled using R 3.6.0 (92). Data were fit using the nls.multstart package (95) to fit sigmoidal uptake curves as described previously (17, 31). Briefly, a nonlinear modeling approach allows the fitting of a 4-point curve using the following equation:

(2) $$y=\frac{a}{1+e^{\frac{x-t_{50}}{w}}}+o$$

The resulting four parameters produced by the fit relate to the amplitude of the curve (*a*), the midpoint of compound depletion from the medium (*t*_50_), the width of the concentration decrease (*w*), and the offset or predicted final value (*o*). The width (*w*) is mathematically defined as the time it takes for the exponent of *e* to go from 1 to −1. This parameter has been modified to depict the time from 10% of substrate utilization [(*a* − *o*) × 0.9] to 90% of substrate utilization [(*a* − *o*) × 0.1] and is depicted in later figures as a 90% usage window. For appearance curves, the signs are changed for a portion of the equation, [−(*x* − *t_50_*)], to invert the model fit. More generally, decreasing and increasing concentrations of compounds in the media were grouped into four different categories: type 1, early depletion or appearance and insufficient data for curve fitting; type 2, sigmoidal fit; type 3, sufficient data but nonsigmoidal shape; and type 4, late depletion or appearance and insufficient data for curve fitting (Fig. S4 to S12).

Curves were visually inspected to ensure that measured data were sufficient and appropriate to fit using this nonlinear function. In some cases, depletion occurred predominantly before or after the sampling interval (types 1 and 4). In these cases, nonlinear depletion curves were not fit since no data were available to indicate a suitable *t*_50_ or the steepness of the curve around this inflection point. Parameters of interest (*t*_50_) and (width) were extracted and used to construct usage window plots to aid in the visualization of substrate depletion preferences and overlapping usage windows. A modified version of the width parameter, 90% usage window, was created by solving the fitted curve for the time of 10% of usage [(amplitude – offset) × 0.9] as well as 90% of substrate utilization [(amplitude – offset) × 0.1]. Following the protocol outlined in the work of Erbilgin et al., the differential of the 4-point sigmoidal fit was used to solve for the slope of all substrate depletion curves (31). The maximum rate was then extracted within the time frame of the experiment (0 to 24 h).

### Determination of substrate specific growth rates of *Paraburkholderia* sp. 1N on targeted substrates.

Specific μ_max_ was determined by culturing on each targeted substrate as the sole carbon source. The medium used for these trials consisted of 1× Wolfe’s vitamin supplement (ATCC MD-VS), 1× Wolfe’s trace mineral solution (ATCC MD-TMS), ammonium chloride (1.5 g/liter), potassium phosphate (0.5 g/liter), and each substrate supplied at an equivalent C content (200 mg of C/liter). *Paraburkholderia* sp. 1N was grown overnight in 10 ml of each sole C medium and then 100 μl was removed into a sterile 2-ml centrifuge tube. Biomass was pelleted at 5,000 × *g* and washed 3 times using fresh medium. The sample was then vortexed, 200-μl subsamples were placed into a 96-well plate, and growth was monitored hourly using a microplate reader at 595 nm (FilterMax F5; Molecular Devices). A total of 3 replicates and an uninoculated blank were used for each sole C medium. Data were imported into R 3.6.0 (92), and the growth rate package (96) was used to fit growth curves for each replicate and extract the average maximum μ_max_.

### Data availability.

The genome assembly for *Paraburkholderia* sp. 1N can be accessed via the NCBI portal using the BioProject accession number PRJNA590275. Draft genome annotation, along with associated material, can be found in KBase (https://narrative.kbase.us/narrative/55022). All LC-HRMS and ^1^H NMR raw data files, associated metadata, as well as processed output have been deposited to the EMBL-EBI MetaboLights database with the identifier MTBLS1692 (https://www.ebi.ac.uk/metabolights/MTBLS1692) (97).

## Supplementary Material

Supplemental file 1
