# Supplementary material for: Ecophysiological Study of Paraburkholderia sp. Strain 1N under Soil Solution Conditions: Dynamic Substrate Preferences and Characterization of Carbon Use Efficiency
Source: Appl Environ Microbiol. 2020 Nov 24;86(24):e01851-20. doi: 10.1128/AEM.01851-20 (PMC7688210; doi:10.1128/AEM.01851-20)
Supplement: Supplemental file 1 [file AEM.01851-20-s0001.pdf]

**Supplemental Material: Ecophysiological Study of *Paraburkholderia* sp. Strain 1N under Soil Solution Conditions: Dynamic Substrate Preferences and Characterization of Carbon Use Efficiency**

K. Taylor Cyle[1], Annaleise R. Klein[2], Ludmilla Aristilde[2,3], Carmen Enid Martínez[1#]

[1]Soil and Crop Sciences, School of Integrative Plant Science, College of Agriculture and Life Sciences, Cornell University, Ithaca, NY, 14853, USA

[2]Department of Biological and Environmental Engineering, Cornell University, Riley-Robb Hall, Ithaca, NY 14853

[3]Department of Civil and Environmental Engineering, McCormick School of Engineering and Applied Science, Northwestern University, Evanston, IL, 60208, USA

**Corresponding Author**

#email: cem20@cornell.edu

**Table S1. SESOM analyte composition as determined by ICP-OES**

| | (mg/L) | | (mg/L) |
|---|---|---|---|
| Al | 1.461 | Mn | 0.104 |
| As | < DL | Mo | < DL |
| B | 0.418 | Na | 5.189 |
| Ba | 1.034 | Ni | 0.005 |
| Ca | 6.089 | P | 1.501 |
| Cd | 0.001 | Pb | 0.019 |
| Co | 0.003 | S | 4.361 |
| Cr | 0.002 | Se | 0.001 |
| Cu | 0.008 | Si | 2.748 |
| Fe | 1.322 | Sr | 0.034 |
| K | 12.671 | Ti | 0.002 |
| Li | 0.004 | V | 0.003 |
| Mg | 1.411 | Zn | 0.952 |

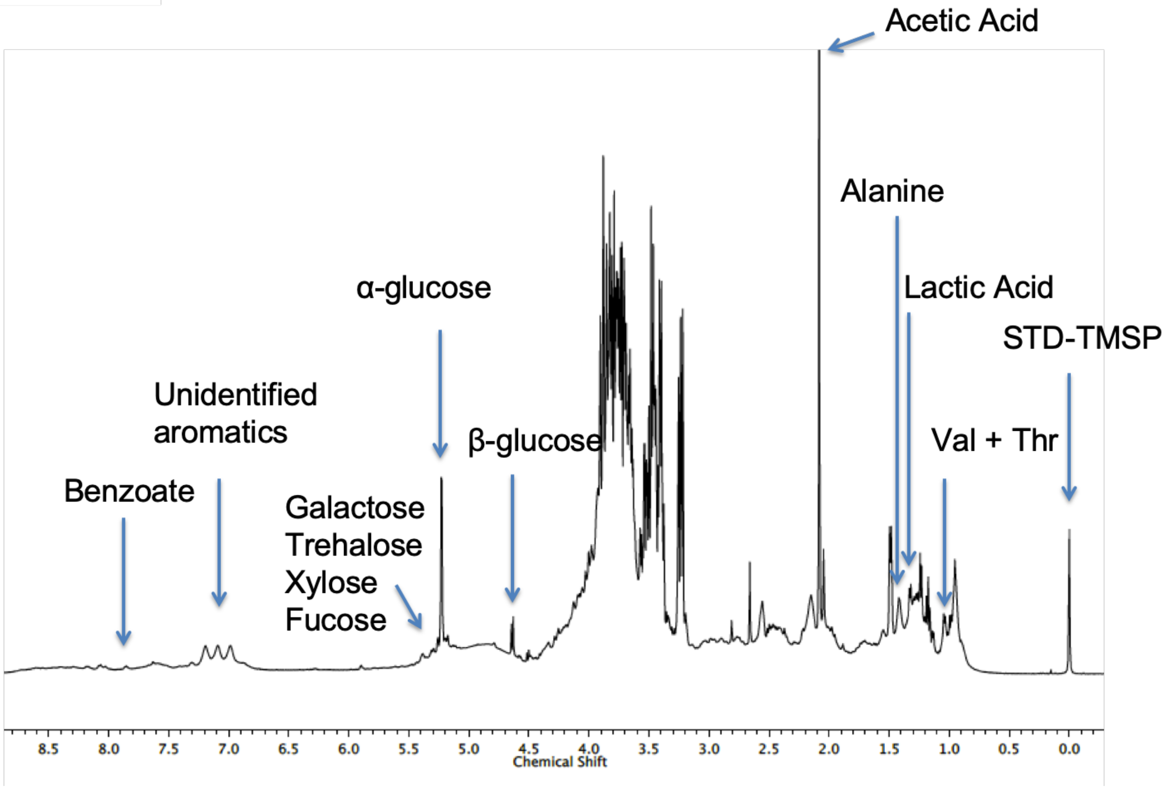


**Figure S1. Example $^{1}$H NMR spectra with peaks of interest annotated.** This specific spectrum is of the initial SESOM solution. See methods for collection and processing parameters.

**Table S2. Assigned NMR resonances in SESOM**

| Compound | Assigned chemical shift (ppm) | Integrated region (ppm) |
|---|---|---|
| Acetate | 2.08 (s) | [2.0601, 2.1] |
| Alanine | 1.49 (d) | [1.46, 1.52] |
| α-Glucose | 5.23 (d) | [5.21, 5.245] |
| β-Glucose | 4.64 (d) | [4.62, 4.66] |
| Lactate | 1.32 (d) | [1.31, 1.34] |
| Unidentified Aromatic | 7.09 (*) | [6.85, 7.35] |

*Three peaks were integrated for this unidentified signal, though they do not represent a triplet.

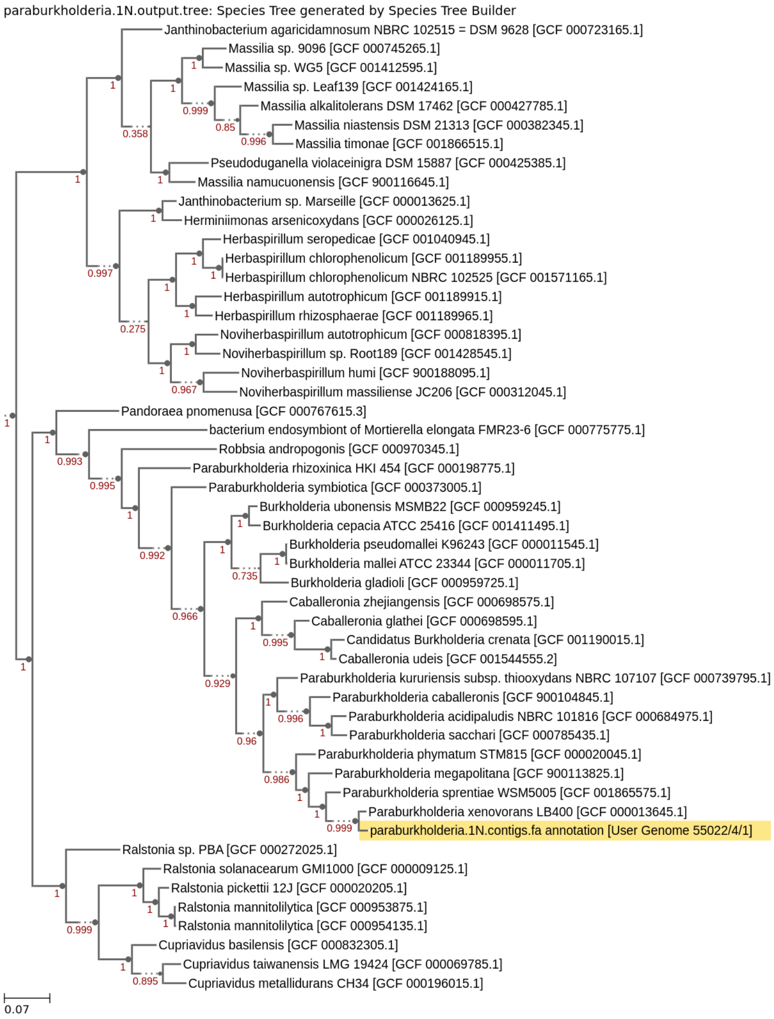


**Fig. S2 Phylogenetic Tree of *Paraburkholderia* sp. 1N in relation to 50 closest genomes.** The tree was created based on a whole genome assembly, annotation, and genomic comparison within KBase using the application 'Insert Set of Genomes Into Species Tree' (v2.1.10), dependent on Fast-Tree2.

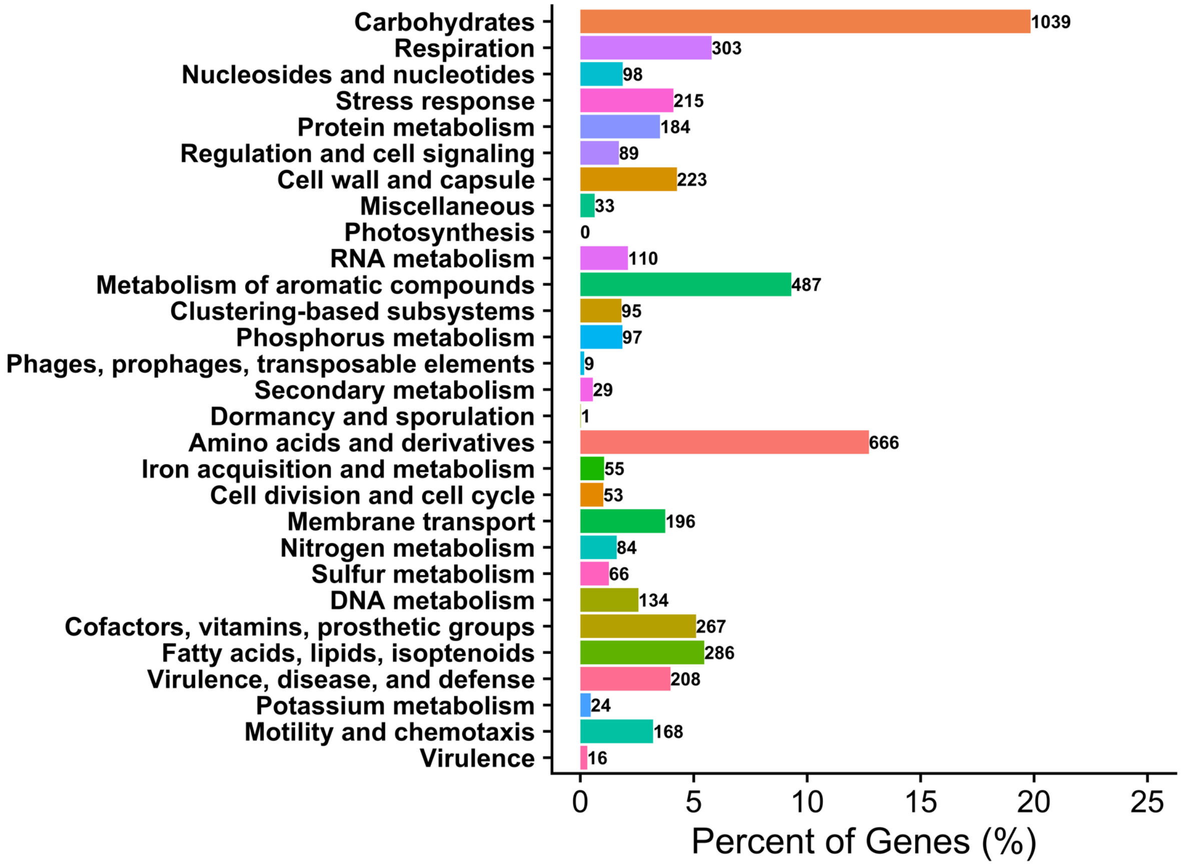


**Fig. S3 RAST Annotated genes from draft genome assembly.** Genomic annotation was conducted using the KBase application "Annotate Microbial Assembly" relying on the RAST annotation toolkit. The annotate genome viewer was used to group annotations based on functional category (see https://narrative.kbase.us/narrative/55022).

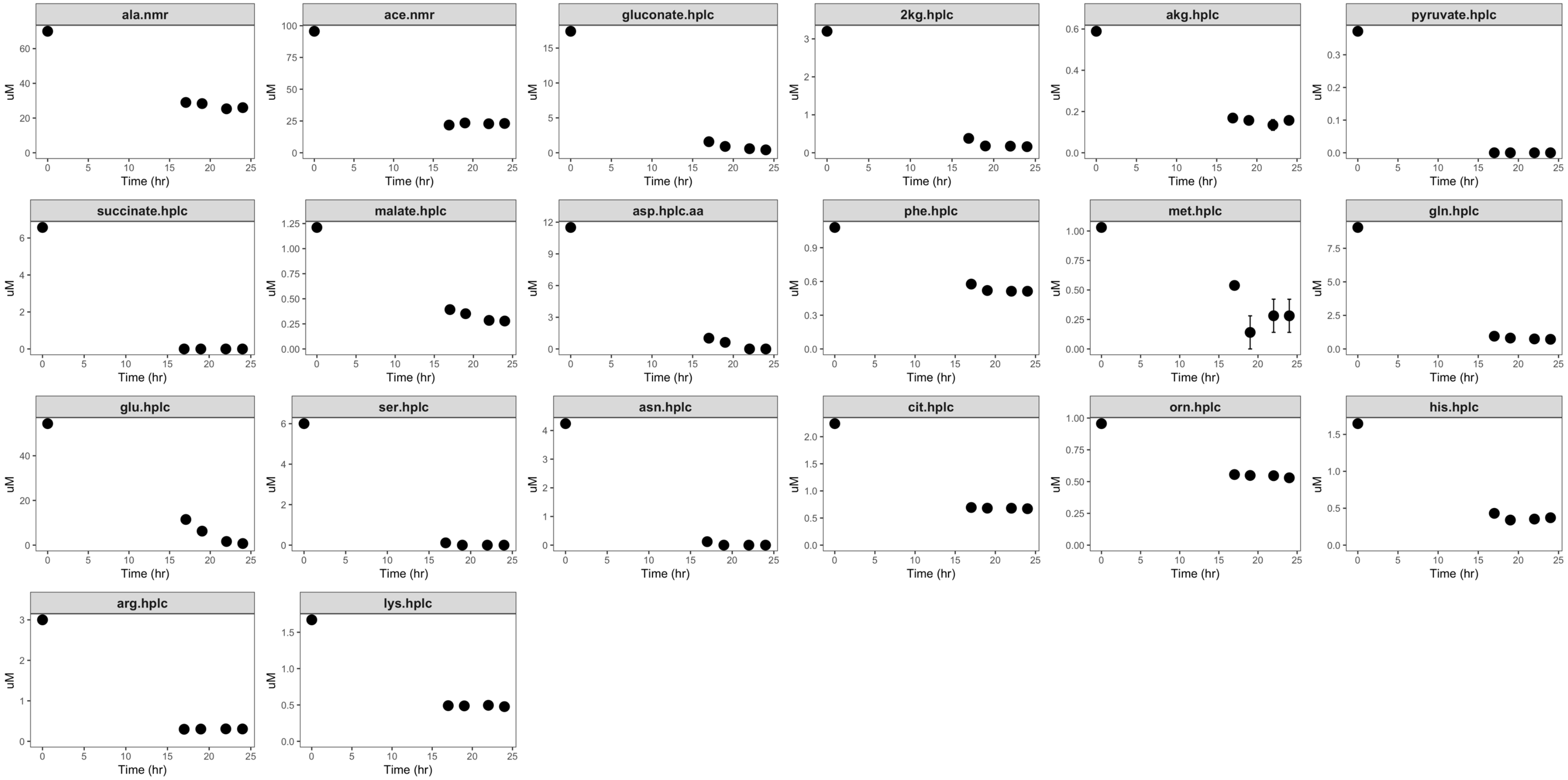


**Fig. S4. Type 1 targeted substrates (n = 20).** The depletion patters of these substrates either did not fit a 4-point sigmoidal pattern or there was insufficient data for curve fitting. In all cases there was still significant depletion and the majority occurred before the first sampling event. Panel headers display the abbreviation for the compound and source of data (hplc = LC-HRMS, nmr = $^{1}$H NMR). Each point represents a mean and error bars indicate standard error (n = 3).

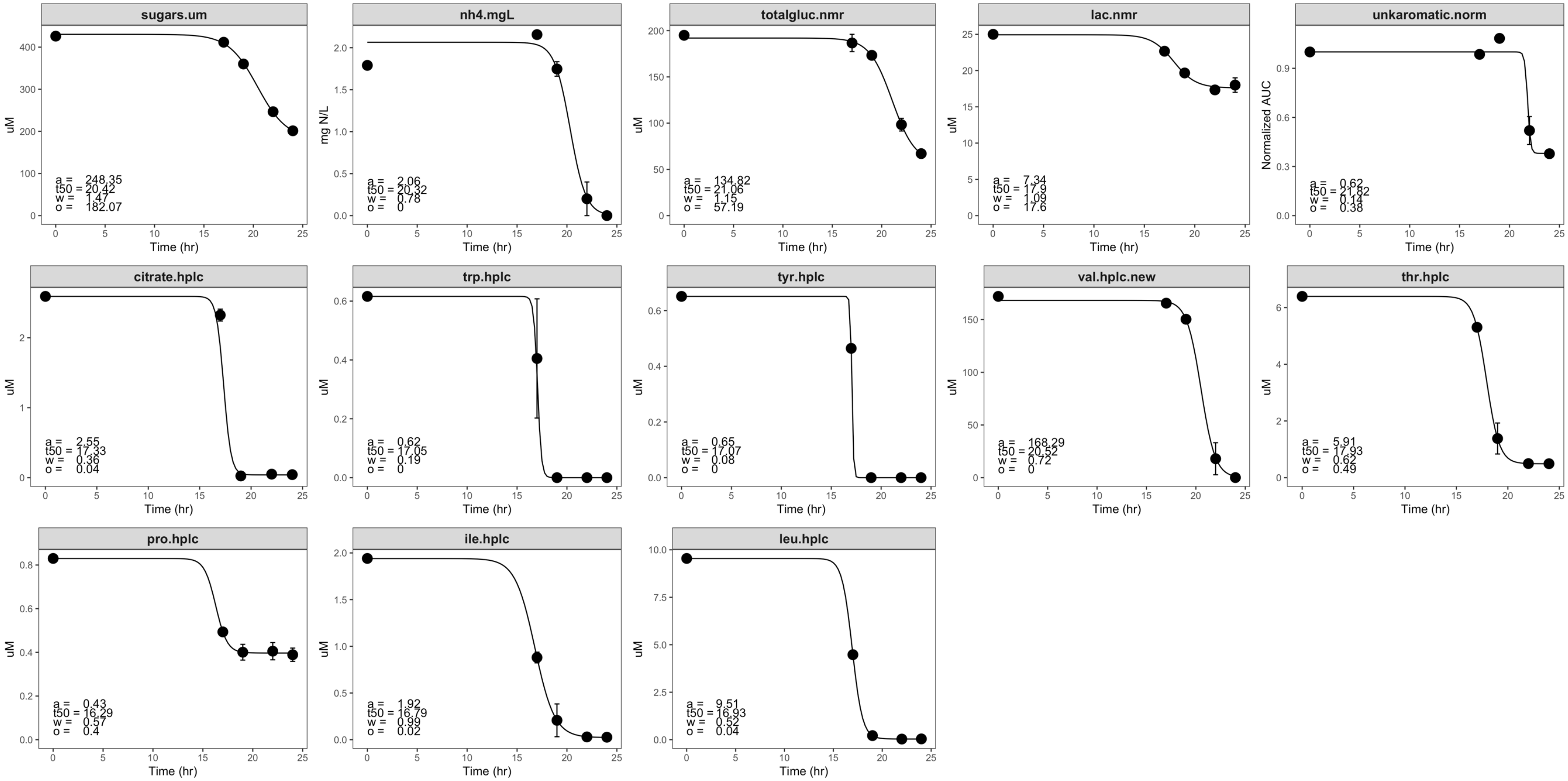


**Fig. S5. Modeled depletion curves for targeted substrates (n = 12).** Model fit parameters are displayed within each panel. Each point represents a mean and error bars indicate standard error (n = 3). Panel headers display the abbreviation for the compound and source of data (hplc = LC-HRMS, nmr = $^{1}$H NMR). The substrate "unkaromatic" is an unidentified signal in the aromatic region of the $^{1}$H NMR spectra (Table S2). It is not included in manuscript figures or the substrate count, but it is included here.

**Untargeted Processing Parameters:**

----------------------------------------------------------------------

XCMSOnline version 2.3.0
XCMS version 1.47.3
CAMERA version 1.26.0

----------------------------------------------------------------------

General parameters
Polarity negative
Retention time format minutes
2. Feature detection
method : centWave
ppm 15
snthr 4
peakwidth 20 60
mzdiff 0.01
prefilter peaks 3
prefilter intensity 100
noise 0
Feature detection results :
2017-12-01_098_18_1X.mzML [B1N-Exometabo-8-11-T0] --> 4819 Features.
2017-12-01_095_15_1X.mzML [B1N-Exometabo-8-11-T1] --> 4534 Features.
2017-12-01_096_16_1X.mzML [B1N-Exometabo-8-11-T1] --> 4327 Features.
2017-12-01_097_17_1X.mzML [B1N-Exometabo-8-11-T1] --> 4493 Features.
2017-12-01_090_12_1X.mzML [B1N-Exometabo-8-11-T2] --> 4493 Features.
2017-12-01_091_13_1X.mzML [B1N-Exometabo-8-11-T2] --> 4474 Features.
2017-12-01_094_14_1X.mzML [B1N-Exometabo-8-11-T2] --> 4435 Features.
2017-12-01_087_9_1X.mzML [B1N-Exometabo-8-11-T3] --> 4280 Features.
2017-12-01_088_10_1X.mzML [B1N-Exometabo-8-11-T3] --> 4472 Features.
2017-12-01_089_11_1X.mzML [B1N-Exometabo-8-11-T3] --> 4473 Features.
2017-12-01_084_6_1X.mzML [B1N-Exometabo-8-11-T4] --> 4209 Features.
2017-12-01_085_7_1X.mzML [B1N-Exometabo-8-11-T4] --> 4165 Features.
2017-12-01_086_8_1X.mzML [B1N-Exometabo-8-11-T4] --> 4354 Features.
3. Retention time correction
method : obiwarp
profStep 0.5
4. Grouping
method : density
bw 5
mzwid 0.025
minfrac 0.5
minsamp 1
5. FillPeaks
6. Diffreport
classes B1N-Exometabo-8-11-T0
classes B1N-Exometabo-8-11-T1
classes B1N-Exometabo-8-11-T2

classes	B1N-Exometabo-8-11-T3
classes	B1N-Exometabo-8-11-T4
statistical test	ANOVA parametric
statistics.threshold.pvalue	0.01
statistics.diffReport.value	into

Finished Running Statistical tests

7. Additional Plots & Statistics
Running mummichog
Printing MDS plot
Printing static PCA and Select Scaling plot

8. Annotation (isotopes & adducts)
featureAnnotation.CAMERA.annotate	isotopes + adducts
featureAnnotation.CAMERA.mzabs	0.015
featureAnnotation.CAMERA.ppm	5
featureAnnotation.CAMERA.sigma	6
featureAnnotation.CAMERA.perfwhm	0.6
featureAnnotation.CAMERA.maxcharge	3
featureAnnotation.CAMERA.maxiso	4
featureAnnotation.CAMERA.intensity	into

9. Putative ID's (METLIN)
identification.METLIN.ppm	15
identification.METLIN.adducts	M-H, M-H2O-H, M+Na-2H, M+Cl, M+K-2H, M+FA-H

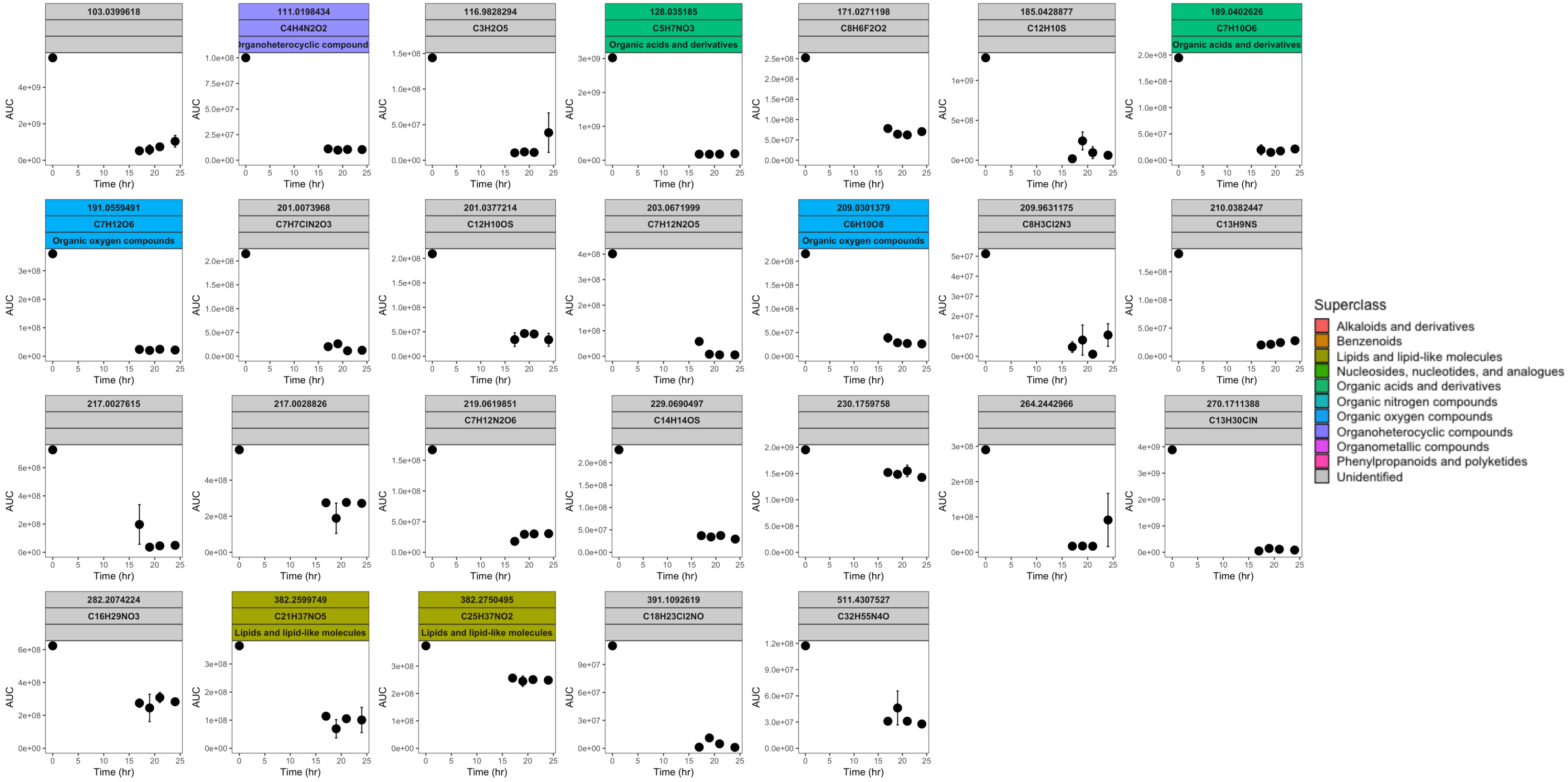


**Fig. S6. Type 1 decreasing features from untargeted LC-HRMS analysis (n = 26).** The depletion patters of these substrates occurred primarily before the first sampling point and therefore their pattern cannot be assessed as sigmoidal or otherwise. Panel headers display the *m/z* of the feature, putatively identified empirical formula, and Superclass. Each point represents a mean and error bars indicate standard error (n = 3).

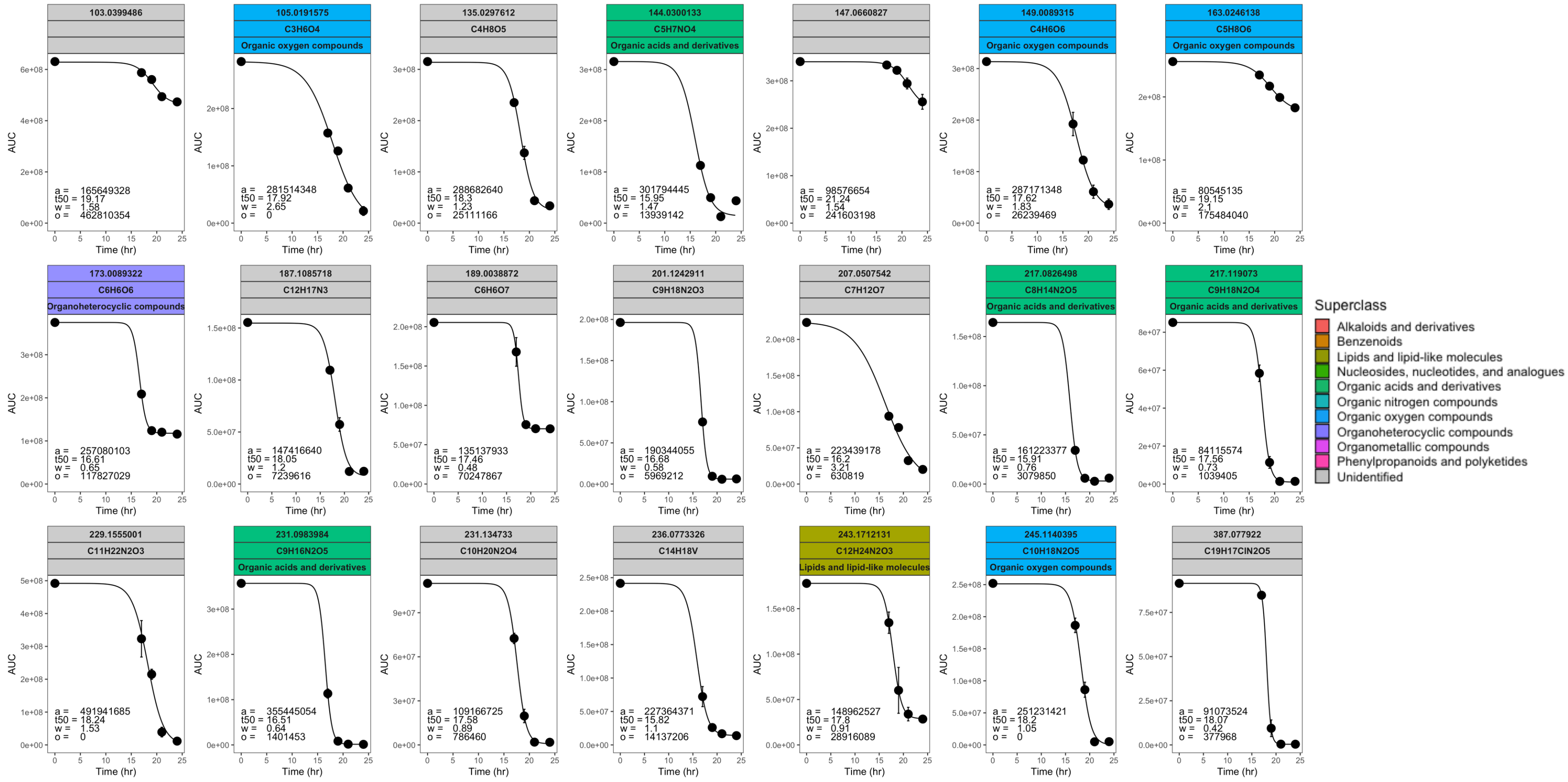


**Fig. S7. Type 2 decreasing features from untargeted LC-HRMS analysis (n = 21).** All depletion patterns could be appropriately fit using a sigmoidal function. Panel headers display the *m/z* of the feature, putatively identified empirical formula, and Superclass. Model fit parameters are displayed within the lower left portion of each panel. Each point represents a mean and error bars indicate standard error (n = 3).

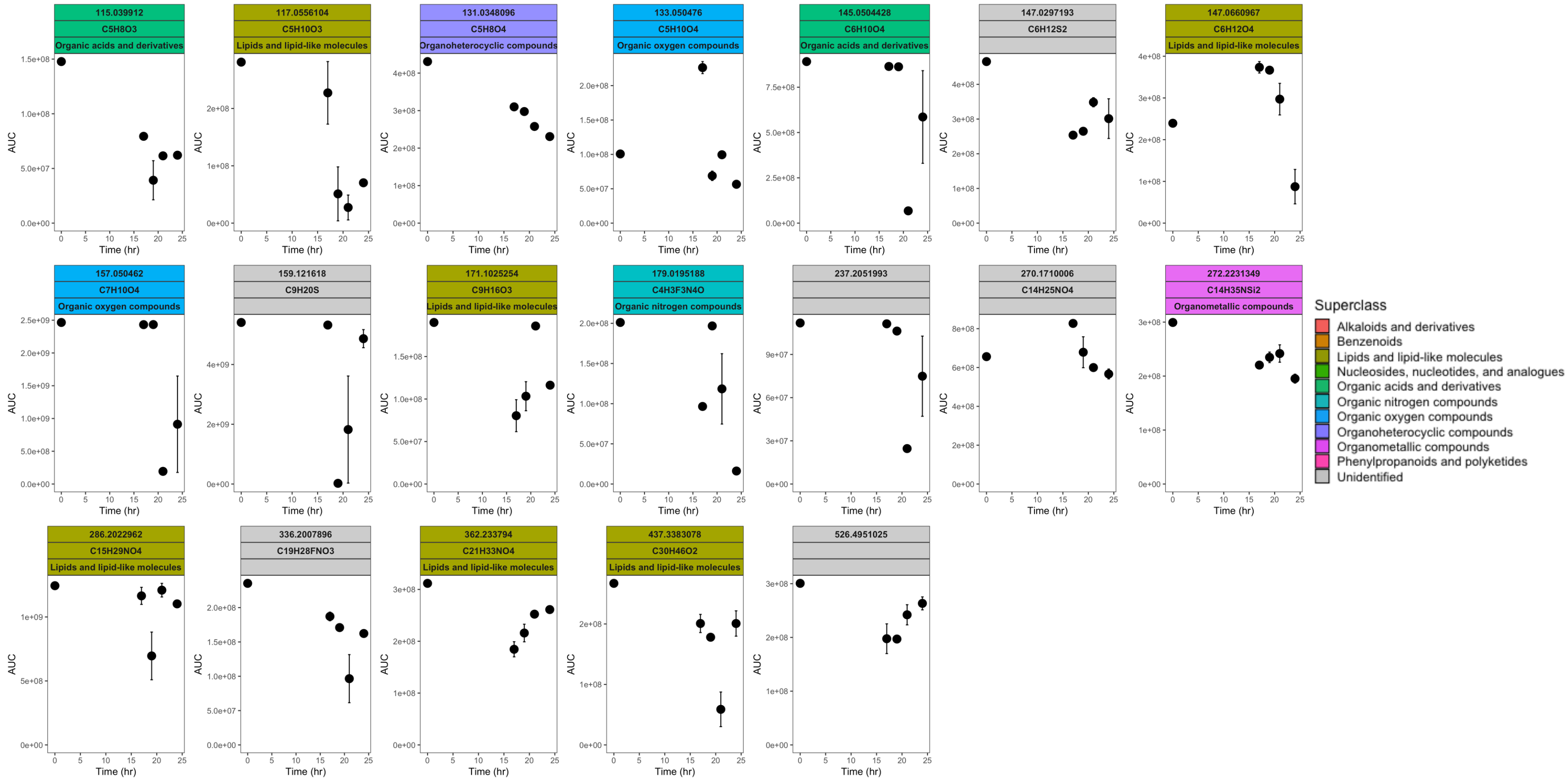


**Fig. S8. Type 3 decreasing features from untargeted LC-HRMS analysis (n = 19).** The depletion patters of these substrates were observable across the timeframe sampled and did not fit a sigmoidal function, though significant depletion was observed between the initial and final time points. Panel headers display the *m/z* of the feature, putatively identified empirical formula, and Superclass. Each point represents a mean and error bars indicate standard error (n = 3).

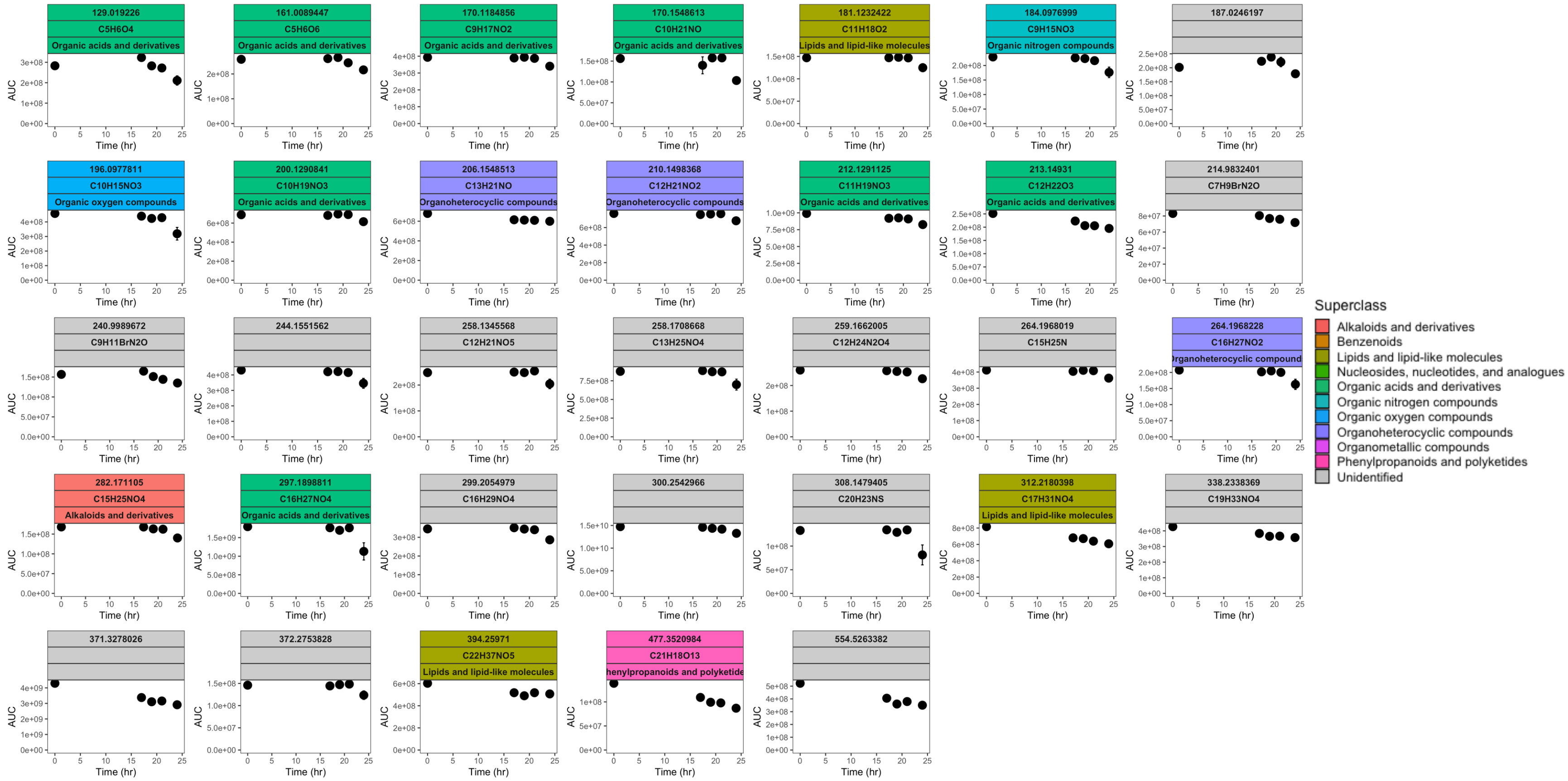


**Fig. S9. Type 4 decreasing features from untargeted LC-HRMS analysis (n = 33).** The depletion patters of these substrates had just commenced during the last sampling point and therefore their patterns cannot be assessed as sigmoidal or otherwise. Panel headers display the *m/z* of the feature, putatively identified empirical formula, and Superclass. Each point represents a mean and error bars indicate standard error (n = 3).

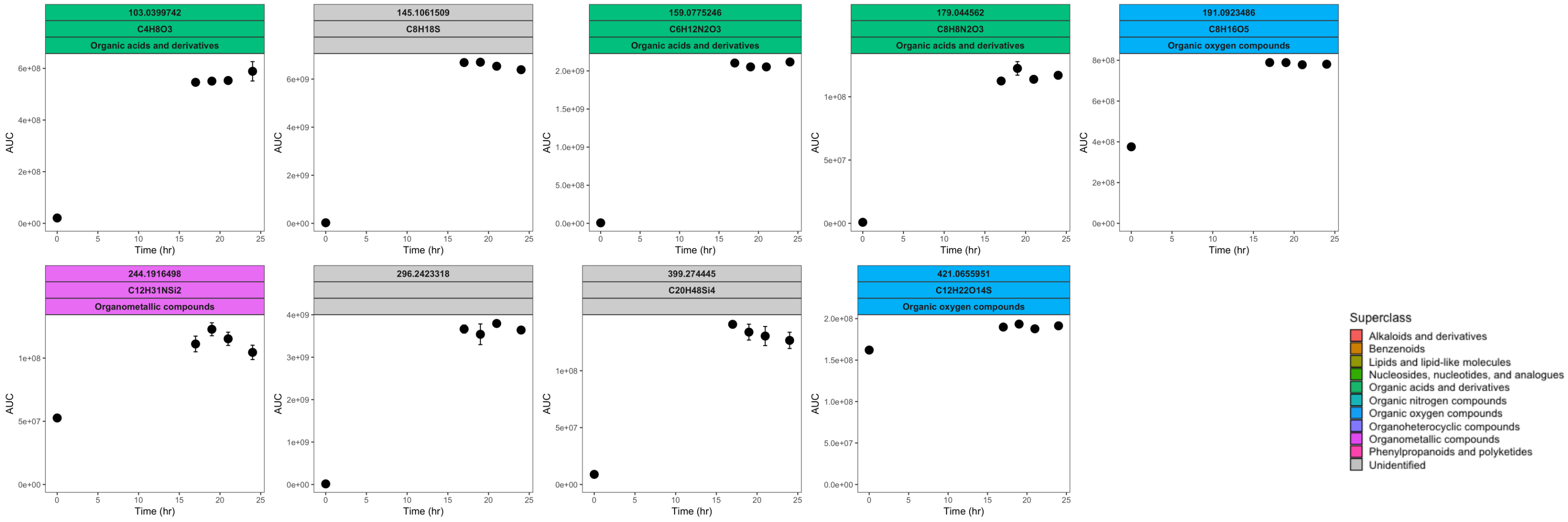


**Fig. S10. Type 1 increasing features from untargeted LC-HRMS analysis (n = 9).** The appearance patters of these substrates occurred primarily before the first sampling point and therefore their pattern cannot be assessed as sigmoidal or otherwise. Panel headers display the *m/z* of the feature, putatively identified empirical formula, and Superclass. Each point represents a mean and error bars indicate standard error (n = 3).

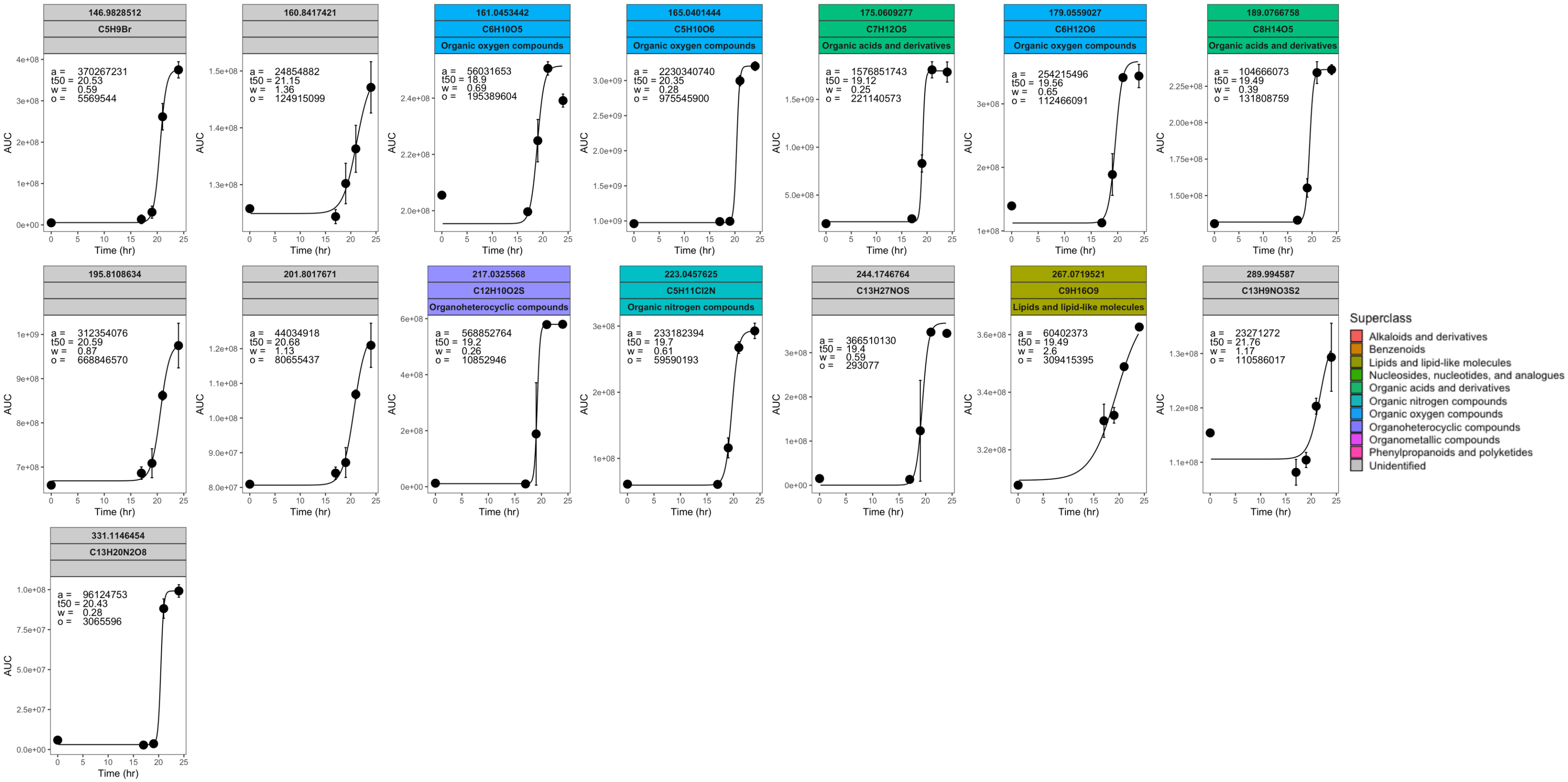


**Fig. S11. Type 2 increasing features from untargeted LC-HRMS analysis (n = 15).** All appearance patterns could be appropriately fit using a sigmoidal function. Panel headers display the *m/z* of the feature, putatively identified empirical formula, and Superclass. Model fit parameters are displayed within the upper left portion of each panel. Each point represents a mean and error bars indicate standard error (n = 3).

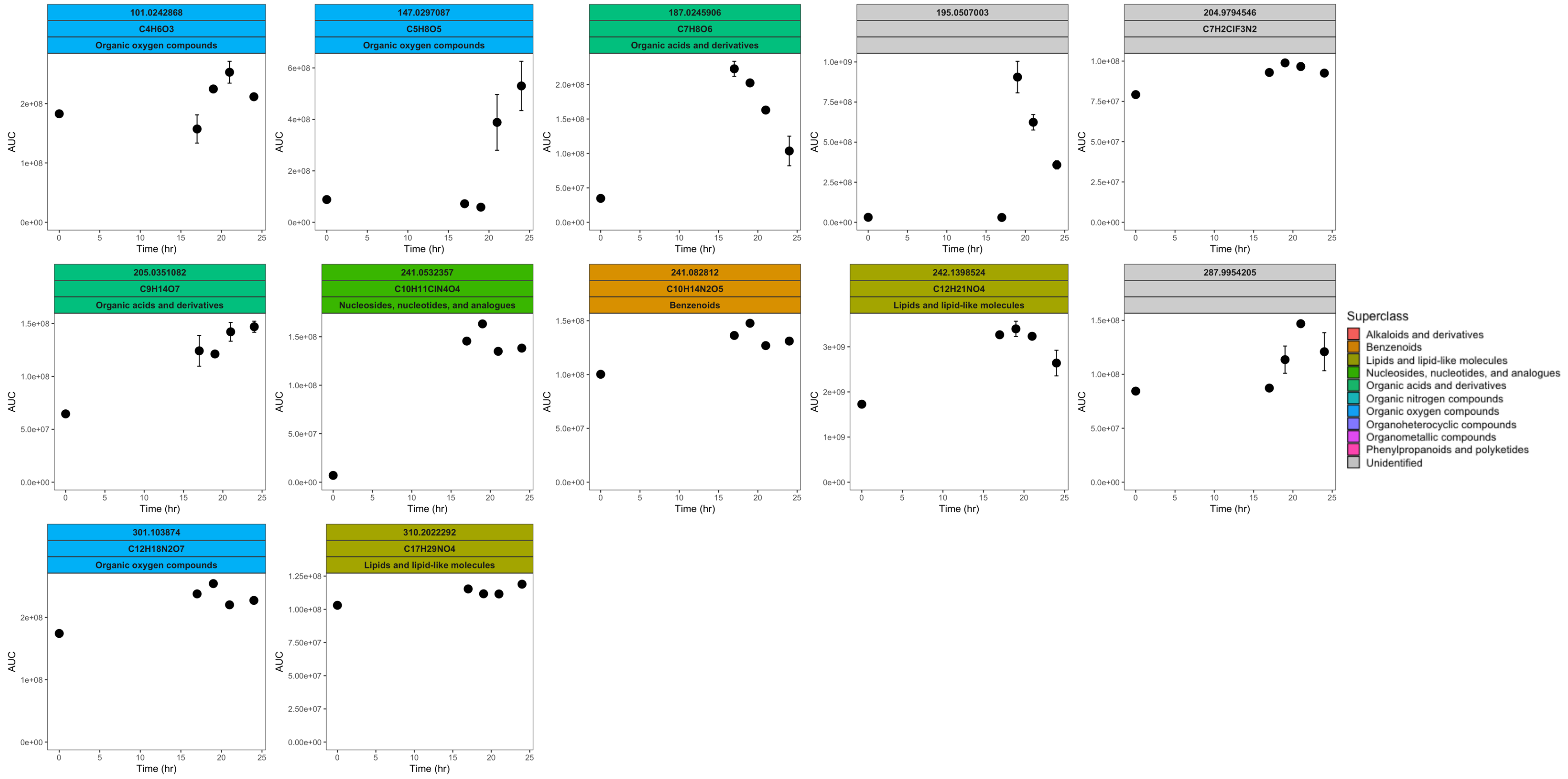


**Fig. S12. Type 3 increasing features from untargeted LC-HRMS analysis (n = 12).** The appearance patters of these substrates were observable across the timeframe sampled and did not fit a sigmoidal function. Panel headers display the *m/z* of the feature, putatively identified empirical formula, and Superclass. Each point represents a mean and error bars indicate standard error (n = 3).
